# Information about historical emissions drives the division of climate change mitigation costs

**DOI:** 10.1038/s41467-023-37130-7

**Published:** 2023-03-14

**Authors:** Alessandro Del Ponte, Aidas Masiliūnas, Noah Lim

**Affiliations:** 1 Department of Political Science, University of Alabama, Marrs Spring Road, Tuscaloosa, AL 35401 USA; 2 Department of Economics, University of Sheffield, 9 Mappin Street, Sheffield, S1 4DT UK; 3 Global Asia Institute, National University of Singapore, 10 Lower Kent Ridge Road, Singapore, 119076 Singapore; 4 Department of Marketing, NUS Business School, National University of Singapore, Singapore, Singapore

**Keywords:** Climate-change mitigation, Climate-change mitigation, Psychology and behaviour, Environmental economics

## Abstract

Despite worsening climate change, the international community still disagrees on how to divide the costs of mitigation between developing countries and developed countries, which emitted the bulk of historical carbon emissions. We study this issue using an economic experiment. Specifically, we test how information about historical emissions influences how much participants pay for climate change mitigation. In a four-player game, participants are assigned to lead two fictional countries as members of either the first or the second generation. The first generation produces wealth at the expense of greater carbon emissions. The second generation inherits their predecessor’s wealth and negotiates how to split the climate change mitigation costs. Here we show that when the second generation knows that the previous generation created the current wealth and mitigation costs, participants whose predecessor generated more carbon emissions offered to pay more, whereas the successors of low-carbon emitters offered to pay less.

## Abstract (web summary)

Who pays for climate change mitigation is an ongoing source of conflict. Here the authors examine how historical carbon emissions influences how much people will pay for climate change mitigation via an economic experiment.

## Introduction

Mitigating climate change is a global social dilemma that spans both geography and, importantly, generations^1^. However, after nearly three decades of diplomatic efforts and 26 UN Climate Change Conferences, countries have failed to reach an agreement that can effectively curb global carbon emissions. Even if countries reached their Nationally Determined Contributions under the Paris Agreement, it would not be enough to keep the global average temperature within 2 °C above preindustrial levels^2,3^. A major hurdle in climate negotiations is the disagreement on how the burden of climate change mitigation should be divided between the industrialized and the developing countries^4^. Developing countries believe that industrialized countries should lead the mitigation effort since they are responsible for the bulk of historical emissions^5^. For example, China supports holding countries accountable for historical emissions both in academic and official communication^6^. Instead, industrialized countries believe that climate change mitigation should be carried out where it is most cost-effective and most beneficial, irrespective of historical emissions^5^. In this paper, we study the nature of this disagreement with an economic experiment. Specifically, we designed an economic game to test whether information about historical emissions^7^ can affect the division of mitigation costs between historically low and high emitters, and assess whether this information influences the success of international climate negotiations.

In our game, participants in the first generation work to develop their country and create wealth for themselves and their successor. However, production also generates exponentially greater emissions and exacerbates climate change. Participants in the second generation inherit the wealth created by their predecessors and negotiate how to divide the mitigation costs to prevent a climate disaster. In this setup, second-generation participants did not personally cause climate change, although they owe their prosperity to their predecessor’s historical emissions. We compare two treatments: in the History treatment, second-generation participants are aware that their current wealth and the climate change problem were created by their predecessor; in the Baseline, participants did not know the origins of climate change or their wealth. The treatment difference reveals how the division of climate change mitigation costs changes in response to accurate information about historical emissions provided by the recent advances in climate science^7^.

By studying how historical emissions affect the division of climate mitigation costs, we illuminate how history shapes efforts for climate change mitigation. Several previous experimental papers studied how climate change negotiations depend on the decisions made by participants in a previous stage^8–12^. However, in previous experiments, the same participants took part in both stages, therefore second-stage decisions would be driven by personal rather than collective responsibility. In our experiment, second-generation decisions are made by a new set of participants who inherit the consequences of the first generation’s decisions. Separating the two generations has an important advantage over previous work: this design reflects more accurately the fact that industrial development in Western countries occurred many decades or even centuries ago, so people alive today did not create them. Instead, if the same participants make both decisions as in prior research, the correlation between decisions in the two stages could be driven by personal characteristics, such as social preferences. Or, the information observed in the first generation could change expectations about the behavior of others, driving decisions in the second generation. The present design overcomes these limitations.

Our study also contributes new insights to the climate change debate on whether countries with high historical emissions should pay for mitigation. On one extreme, there is the view that countries that created the bulk of carbon emissions are morally responsible for the actions of their predecessors and should cover most of the mitigation costs^13–15^. Intermediate proposals balance historical emissions with the present capacity to pay: the principle of common but differentiated responsibilities in the light of different national circumstances^16,17^. On the other side, the notion of historical responsibility is rejected for a number of reasons, including that responsibility is personal^18^, that previous generations simply did not know that carbon emissions could cause disastrous climate change^19,20^, or that establishing historical responsibility is an impossible task due to practical constraints^21,22^. While we acknowledge this debate, we remain agnostic about which side is right, and focus our attention on the effect of information about historical emissions on the share of climate change mitigation costs that countries are willing to bear.

Next, we illustrate the design of the climate game. We designed a game in which higher production by the first generation increased both the wealth and the costs that the next generation needed to pay to avert a climate disaster. Two participants (denoted as A1 and B1) were the first-generation leaders of two fictional countries A and B. Two other participants (A2 and B2) were the leaders of these same countries in the second generation. The first generation never interacted with the second generation.

First-generation leaders could create wealth at the expense of environmental costs by completing real effort tasks (moving up to 40 sliders from a random starting position to the middle). Earnings for completing each task (piece rates) varied across pairs: there were equal probabilities that the piece rates would be equal ($8 per task for A1, $8 for B1), moderately unequal ($10 for A1, $6 for B1), or highly unequal ($12 for A2, $4 for B1). We used three sets of piece rates to create heterogeneity in emissions and wealth in the second generation. In addition to generating income, first-stage production increased the climate change mitigation costs that the second generation would need to pay to avoid a climate disaster. In line with the literature^23,24^, the costs were convex in production (see Methods for more details).

**Fig. 1:**
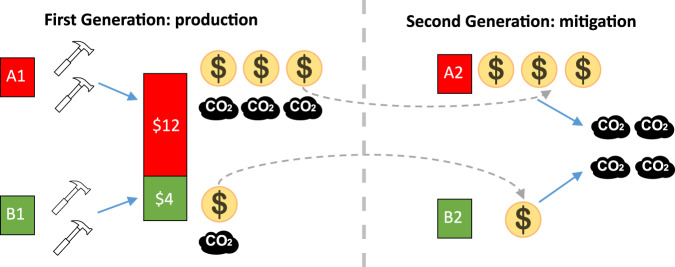
Illustration of the game structure. Participants A1 and B1 provide effort at a certain piece rate (in the example, A1 earns $12 per task and B1 earns $4), creating wealth and emissions. Participants A2 and B2 inherit the wealth of their predecessors and use it to mitigate climate change.

In the second generation, A2 inherited the wealth of A1 and B2 inherited the wealth of B1: the starting endowment of A2 (B2) was equal to the total earnings of A1 (B1), plus $600. Then, A2 and B2 decided how to divide the costs of mitigating climate change to prevent a disaster that could destroy their earnings (for an illustration, see Fig. 1). Costs were divided using an ultimatum game with the strategy method. The proposer made an offer on how to split the costs and the responder set the maximum amount they were willing to pay. The proposer’s offer was implemented if it was acceptable to the responder; otherwise, it was rejected. If the offer was rejected, there was a 90% chance that participants will earn nothing because of a climate disaster and a 10% chance that the participants would keep their original endowments. Each participant made decisions both as a proposer and as a responder and their role was determined by a random draw once all data had been collected. Second-generation participants played the game four times, using the outcomes from four different first-generation pairs. Multiple observations allow us to classify each participant based on their sensitivity to the outcomes from the first generation.

We compared choices in two treatments. In the History treatment, second-generation participants were fully informed about how their endowments and climate change mitigation costs were determined, as well as the choices that the first generation made. In the Baseline, no information about the first generation was provided; instead, participants were seeded with the same outcomes (endowments and costs) as the participants in the History treatment but were not informed about the origins of these variables. This design allows us to identify the effect of information about historical emissions independent of the emissions and wealth generated by the first generation. We recruited 103 participants for the first generation, 101 participants for the second generation in the History treatment (62% female; average age: 22 years old), and 103 participants for the second generation in the Baseline (69% female; average age: 22).

We now present the game-theoretic predictions. Equilibrium predictions are identical in the History treatment and the Baseline because information about the origins of climate change is irrelevant for incentives. In section S3 of the Supplementary Information, we show that for the parameters used in the experiment, there always is a Nash equilibrium in which the proposer and the responder reach an agreement. Any division of costs can be supported by some Nash equilibrium, as long as both players earn more than they would if no agreement was reached. If climate change mitigation costs are sufficiently high, there also are equilibria in which no agreement is reached, in addition to the equilibria in which agreements are successful.

An alternative hypothesis is that information about historical emissions from the previous generation evokes a sense of collective guilt and makes the descendants of high emitters more willing to pay for climate change mitigation. In our setup, greater production by A1 benefits A2, but it also increases the mitigation costs and potentially hurts B2, which might make A2 feel guilty. Guilt is relieved by making amends^25^; A2 could do so by offering to pay more as a proposer (which decreases the disaster risk and increases the earnings of B2 if disaster does not occur) or by accepting lower offers as a responder (which decreases the disaster risk). Since the wrongdoer (A1) is not the same person as the one who can make amends (A2), the decisions of the second generation would be driven by collective, rather than personal guilt^25^. Previous work found that collective guilt may occur even in minimal intergroup situations^26^ and can drive the willingness to mitigate climate change^25^.

Our design models several features that define collective guilt in climate negotiations: Current leaders share the identity with their predecessors (reinforced in the experiment using the framing); are temporally separated from their predecessors; and personally benefit but also face a greater risk from their predecessor’s emissions. These elements of shared identity, temporal separation, personal benefit, and greater risk make collective guilt interesting to study empirically because they are the distinctive elements of the global climate mitigation dilemma.

Lastly, we provide an overview of the main variables. We designed the experiment to identify whether participants who inherit more carbon emissions cover a larger share of climate change mitigation costs in the History treatment. Two key variables are therefore the stock of historical emissions that second-generation participants inherit and the share of costs that second-generation participants cover.

Historical emissions are defined as the share of climate mitigation costs created by the participant’s predecessor. We measured historical emissions on a scale from 0 to 1, calculated as a ratio of costs created by the participant’s predecessor to the total costs created by both first-generation participants (see Supplementary Fig. A1). Second-generation participants were exogenously assigned to a combination of inherited wealth and historical emissions, which differed across participants due to differences in production and productivity by first-generation participants. Supplementary Fig. A2 shows the distribution of historical emissions to which participants could be assigned. Heterogeneity in historical emissions allows us to identify how it affects climate negotiations.

The share of costs that participants covered is the main outcome of climate negotiations. We calculate it by matching the proposers to all the responders who were assigned the same first-generation outcome and calculating the share of costs that the proposer would pay conditional on the offer being accepted; in other words, the share of costs is calculated as the average proposer’s offer, weighted by the acceptance probability. On average, proposers paid 54% of the costs in the History treatment and 51% in the Baseline. We also found that 60% of participants reached a successful agreement in the History treatment and 61% did in the Baseline, a difference that is not statistically significant (Mann–Whitney *U* test two-sided *p* = 0.95). As a robustness check, we also used the individual decisions of the proposers and responders, even if they did not reach an agreement.

## Results

### Cost division

**Fig. 2:**
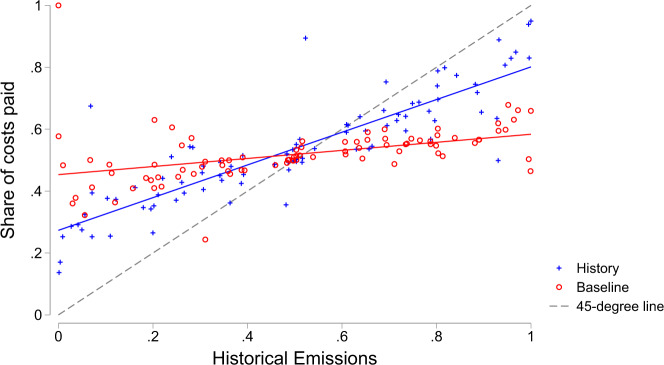
Share of the climate mitigation costs paid by the proposer in the History treatment (blue crosses) and in the Baseline (red circles), across different levels of the proposer’s historical emissions. Each marker represents a different outcome created by a first-generation pair of participants. Blue and red solid lines indicate the best linear fit for the data; the 45° line indicates how costs would be divided if costs were always divided proportionally to historical emissions.

First, we study how the second-generation participants who successfully reached an agreement divided climate change mitigation costs. Figure 2 shows the share of costs paid by the proposer in each treatment. Each marker represents an outcome from the first generation. For each outcome, we calculated the expected division of costs by aggregating the decisions of all proposers and responders assigned to that outcome. We find that the share of costs covered by participants was correlated with historical emissions more strongly in the History treatment than in the Baseline (Pearson’s *r* = 0.86 in the History treatment, *r* = 0.42 in the Baseline).

Table 1 shows that these results hold in OLS regressions where the dependent variable is the share of costs that participants paid. Since the data is aggregated on the first-generation outcome level, we do not have repeated observations and therefore we use an OLS regression with heteroscedasticity-robust standard errors calculated using the HC3 bias correction method. In the History treatment, participants with greater historical emissions covered a larger share of the costs ($\hat{\beta }=0.53$ in Model 1). In the Baseline, the stock of historical emissions also has a small but positive effect ($\hat{\beta }=0.13$ in Model 2). Model 3 shows that historical emissions had a stronger effect in the History treatment than in the Baseline ($\hat{\beta }=0.40$). Model 4 includes both historical emissions and relative wealth, which are correlated and could potentially explain why participants pay more for climate change mitigation. When we account for relative wealth, historical emissions remain significant in the History treatment but are no longer significant in the Baseline. It indicates that participants who unknowingly inherited greater emissions paid more because they received higher endowments. Next, we study the decisions of both proposers and responders, regardless of whether they led to an agreement or not. Since each participant made four decisions in each role, we account for the dependence using a random-effects GLS regression with a random effect for each participant and standard errors clustered on the participant level. We find that in the History treatment, historical emissions increase both the offers of the proposers and the willingness to pay of the responders (Supplementary Table A1 and Supplementary Table A2). In the Baseline, the effect of historical emissions is weaker and disappears once differences in wealth are taken into account. The results do not change if we additionally control for age, gender, political orientation, or the support for action against climate change (Supplementary Table A13 and Supplementary Table A14).

**Table 1:** OLS regression of the share of costs that the proposer paid conditional on reaching the agreement

	(1)	(2)	(3)	(4)
	H	B	H and B	H and B
Historical emissions	0.53***	0.13**	0.13**	0.11
	(13.49)	(2.80)	(2.79)	(0.74)
Total costs	−0.018	−0.037	−0.027	−0.027
	(−0.78)	(−1.18)	(−1.42)	(−1.37)
History treatment			−0.18***	−0.18***
			(−4.71)	(−4.70)
History × Emissions			0.40***	0.40***
			(6.50)	(6.46)
Relative wealth				0.17
				(0.20)
Constant	0.28***	0.47***	0.47***	0.40
	(9.92)	(10.16)	(12.17)	(1.20)
Observations	90	87	177	177
*R*^2^	0.758	0.199	0.647	0.647

*t* statistics in parentheses; costs are measured in thousands.

The dependent variable is the average share of costs that the proposer paid, aggregated across all the decisions made by the proposers and responders who were assigned to the same first-generation outcome. The number of observations is therefore equal to the number of first-stage outcomes for each player type in that treatment. Historical emissions are calculated as a ratio of climate mitigation costs created by the proposer’s predecessor to the total climate mitigation costs created by the first generation. Climate costs indicates the total climate mitigation costs created by the first generation (in thousands of experimental dollars). History × Emissions is the interaction between historical emissions and the History treatment variable. Relative wealth is calculated as the proposer’s endowment, divided by the sum of endowments received by both participants. H denotes the History treatment; B denotes the Baseline. There were 47 first-stage outcomes and two player types in generation 2 (A2 and B2), but in some of the 94 distinct outcomes, the proposer’s offer was never accepted, leaving 90 observations in the History treatment and 87 in the Baseline. A conservative HC3 bias correction method was used to calculate heteroscedasticity-robust standard errors. No adjustments were made for multiple comparisons.

*p < 0.05, **p < 0.01, ***p < 0.001.

### Classification of strategies

To better understand how participants respond to information about history, we classified the strategy of each participant by regressing their four decisions on historical emissions. We classified the strategy as proportional to emissions if the estimated regression coefficient was close to 1 (i.e., between 0.95 and 1.05). In contrast, we classified the strategy as orthogonal to emissions if the coefficient was close to 0 (i.e., between −0.05 and 0.05), which indicates that a participant disregarded historical emissions. We find that 22% of proposers divided costs proportionally to emissions in the History treatment, but none did so in the Baseline (Supplementary Fig. A3). Instead, 20% of proposers disregarded the stock of historical emissions in the Baseline, while only 7% did so in the History treatment (the same pattern holds for the responders; see Supplementary Table A4 for full results). The results are similar if we use a wider interval to classify strategies (±0.1 or ±0.2, instead of ±0.05; see Supplementary Table A4): in both cases, more participants are classified as using a proportional strategy and fewer disregard historical emissions in the History treatment than in Baseline. The cumulative distribution functions of the individually estimated regression coefficients were significantly different between the two treatments (two-sample Kolmogorov-Smirnov test, approximate asymptotic two-sided *p* < 0.001 for both proposers and responders; see Supplementary Fig. A4 for further details), indicating greater use of strategies accounting for emissions in the History treatment.

### Heterogeneous treatment effects

For policy purposes, it is important to understand how the information about history affects the willingness to pay for climate change mitigation, especially for participants who inherited more historical emissions. Thus, we split participants depending on whether their predecessors created more or less than half of the climate change costs. On average, proposers with greater historical emissions offered to pay a significantly higher share of the costs in the History treatment than in the Baseline (61% of the costs in the History treatment, compared to 53% in the Baseline; see Supplementary Fig. A5). The result is reversed for proposers who inherited fewer emissions, who agreed to cover up to 36% of the costs in the History treatment, compared to 44% in the Baseline. Both differences are statistically significant (see a GLS regression in Supplementary Table A3). Participants who inherited more emissions offered to pay more when they were informed about the historical emissions (model 3 in Supplementary Table A3), but they did not change their willingness to pay as responders (model 4 in Supplementary Table A3). The results are reversed for participants with less historical emissions, who were willing to pay less both as proposers and as responders, when informed that their predecessor created the emissions (models 5 and 6).

### Ex-post rationality

Consistent with the results in previous ultimatum game experiments^27–29^, we find that the costs were divided evenly (proposers on average covered 53%). Therefore, we tested whether the offers of the proposers were ex-post rational. For each combination of wealth and climate change mitigation costs created by the first generation, we calculated the optimal proposer’s offer given the acceptance thresholds of all potential responders. We find that proposers in the History treatment would maximize their payoffs by dividing the costs almost proportionally to their historical emissions, but in the Baseline, the ex-post rational strategy is to divide the costs much more evenly (Supplementary Fig. A6). On average, chosen offers were close to ex-post rational offers, indicating that proposers maximize their earnings by taking historical emissions into account in the History treatment but not in the Baseline (Supplementary Fig. A7).

### Obstacles to reaching an agreement

We expected that agreements would be more difficult when participants needed to cover a larger amount of costs and when they were further apart in terms of wealth or historical emissions. We tested these predictions by regressing the expected probability of agreement on the inequality in wealth and in historical emissions, and controlling for the total amount of costs. We find support for these predictions: agreements were less likely when there was greater inequality in historical emissions ($$\hat{\beta }=-0.35$$, $$p=0.036$$; Model 1 in Supplementary Table A5) and inequality in wealth ($$\hat{\beta }=-2.44$$, $$p=0.01$$; Model 2). The effect of inequality was the same in the History treatment as in the Baseline. We also tested whether participants were less likely to agree if their ancestors were further apart in terms of productivity. Compared to participants assigned to the first-stage outcomes with equal productivity (i.e. $8 per task for both A1 and B1), those with moderately unequal piece rates ($10 for A1 and $6 for B1) and highly unequal piece rates ($12 for A1 and $4 for B1) were just as likely to reach an agreement (moderately unequal: $$\hat{\beta }=-0.05$$, $$p=0.37$$; highly unequal: $$\hat{\beta }=-0.12$$, $$p=0.052$$; Model 3 in Supplementary Table A5). We conclude that reaching an agreement was more difficult when participants had to cover greater climate mitigation costs and when there was greater inequality in wealth or historical emissions, but not in productivity in the previous generation.

### Additional baseline treatment

In the original Baseline treatment, we did not inform second-generation participants about the origins of the climate change mitigation costs. We did so to obtain a clean comparison of having full information about the predecessors’ actions in one treatment, and no information in the other. However, second-generation participants might behave differently only because they believe that the seeded numbers were generated by the computer or chosen by the experimenter. To exclude this possibility, we ran an additional baseline, in which second-generation participants (*n* = 93, average age 22.9 years, 57% female) were seeded the same first-generation outcomes as the participants in the original baseline. In this new baseline (which we call Baseline with Predecessors), participants were informed that their endowments and climate change mitigation costs were determined by their predecessors who took part in an earlier experiment. We framed this treatment similarly to the History treatment, describing the predecessors as earlier leaders of countries A and B. However, participants did not know what game was played, what decisions the first-generation participants made, and how these decisions affected mitigation costs and endowments.

Supplementary Tables A7–A12 illustrate the results where the original Baseline is replaced by the Baseline with Predecessors, replicating Tables 1 and A1–A5. We replicate the result that historical emissions have a stronger effect on cost division in the History treatment than in the Baseline with Predecessors (the interaction term in Model 3 of Supplementary Table A7 is $\hat{\beta }=0.31$, $p \, < \, 0.001$). The size of the effect is smaller than when the original Baseline is used (the interaction term is $\hat{\beta }=0.40$ in Table 1), which is driven by a larger coefficient of historical emissions in the Baseline with Predecessors ($\hat{\beta }=0.22$) than in the original Baseline ($\hat{\beta }=0.13$ in Model 2, Table 1), although the difference between the two is not significant ($p=0.087$; statistical significance is calculated by pooling Models 2 from Table 1 and A7 and estimating the interaction between a treatment dummy and historical emissions; see Supplementary Table A15). The difference between the two baselines remains insignificant when we disaggregate the data to look at the effect of historical emissions on proposers’ offers ($p=0.068$ in Supplementary Table A15) or on responders’ maximum willingness to pay ($p=0.225$ in Supplementary Table A15). We also replicate the finding that historical emissions have a stronger effect on the decisions and the division of costs in History than in Baseline with Predecessors, even when controlling for relative wealth (Model 4 in Supplementary Table A7 and Model 5 in Supplementary Table A8 and Supplementary Table A9).

Overall, we replicate the result that the effect of historical emissions has a stronger effect in History than in the Baseline, even when participants are informed about the existence of their predecessors. The differences between the two baseline treatments are not statistically significant, although the estimated coefficient for cost division is higher in the Baseline with Predecessors.

Finally, similar to the original Baseline, when using the Baseline with Predecessors, we found that the History treatment did not change the probability of reaching an agreement. However, in the Baseline with Predecessors, the coefficients for inequality in emissions and wealth were not significant, indicating that more inequality did not reduce the probability of a successful agreement (Supplementary Table A12).

## Discussion

We found that when participants with greater historical emissions were informed about it, they offered to pay a larger share of climate mitigation costs. This behavior is consistent with collective responsibility^30,31^, even though the generation that pays for climate change mitigation and the generation who created climate change never interacted. Our findings suggest that citizens in developed countries may accept paying more for climate change mitigation if the link between past emissions, present wealth, and present climate costs is made clear to them. Recent advances in climate science enable policymakers to communicate this information with greater accuracy than ever before^7^.

Our experiment also shows that developed countries might have to offer paying for a larger share of climate change mitigation costs because failure to do so reduces the probability of reaching an agreement. In the History treatment, which models the current state of scientific knowledge about historical emissions, proposers would maximize their earnings by proposing to divide the costs proportionally to historical emissions, because responders would reject offers that ignore historical emissions. By securing a climate agreement that averted disastrous climate change, proposers were better off because the decreased incidence of disasters outweighed the increased payments for climate change mitigation.

A possible interpretation of these findings is that in the History treatment, information about the predecessor’s carbon emissions provided a salient cue for the fraction of costs that the participant should cover. This cue, which was unavailable in the Baseline, might act as a focal point or an anchor, which are known to affect decisions^32–34^. Salience might be especially important because there are multiple Nash equilibria in the game. Future work could study whether information about historical emissions affects decisions because of salience and whether decisions would be affected by other kinds of salient information, including irrelevant anchors.

Although information about historical emissions significantly affected offers, the probability of reaching a climate agreement was the same regardless of whether participants were informed about historical emissions. The reason is that in the History treatment, the successors of high emitters made higher offers, whereas the successors of low emitters made lower offers. Thus, the combined effect of more generous offers by the successors of high emitters and lower offers by the successors of low emitters resulted in similar average offers and similar agreement rates in the History treatment and in the Baseline. In these two conditions (but not in the Baseline with Predecessors), we also found that participants in the second generation were most successful in reaching an agreement when they were bequeathed with similar wealth and historical emissions. This finding complements previous results about how international inequality thwarts efforts to curb climate change^35–38^ by showing that international cooperation would have been easier if inequality between countries had been lower.

By using different participants to model each generation, the present experiment overcomes key limitations of previous work that studied the role of climate responsibility. For example, in previous studies, the same participants played both stages of the game^8,11,12^; climate responsibility was not studied in a social dilemma game^39^; or the focus was on the creation of climate costs, not their mitigation^40^. Accordingly, our results are different. For example, we find that information about the predecessor’s responsibility for generating the emissions did not reduce the probability to reach an agreement, in contrast to a previous finding that participants were reluctant to cooperate with those who created higher thresholds in the first stage^12^. In our experiment, motives for negative reciprocity are eliminated because the second generation is not personally responsible for the first-stage outcomes.

Our study has several limitations that could be addressed in future work. For simplicity, the experiment involved only two participants per generation, modeling two large economies with different historical emissions and wealth (e.g., the United States and Brazil). If the game involved a larger number of players, the results might be different, for instance due to diffusion of responsibility^41,42^. We also used a one-shot negotiation game, and it would be interesting to see if the results would change if participants could adjust their offers over multiple rounds. Future research could also use real national identities instead of fictional ones. Previous research has shown that national identity can influence attitudes toward globalization^43^ and international solidarity^44^. It would be interesting to study whether national attachments would alter people’s willingness to pay for ameliorating the climate wrongs committed by their co-national ancestors.

Finally, in the present experiment, we made several assumptions that future work can relax. For instance, we assumed that first-generation participants knew that production generates carbon emissions that the future generation will have to mitigate. Only very recent generations meet this knowledge condition^21^, so future work could test whether information about historical emissions affects willingness to pay for climate change mitigation even if participants know that their predecessors were unaware of the consequences of their production. Also, we assumed that the historical contribution to climate change can be identified from the information about the total historical emissions of each country. However, the effects of historical emissions may be nonlinear and depend on when and where they were released^22^. Countries’ contribution to the climate change problem would therefore depend not just on the total emissions, thus making historical responsibility more difficult to establish. We also made the simplifying assumption that climate change negotiations can either succeed or fail in avoiding catastrophic climate change. In practice, countries might reach a partial agreement that would reduce but not eliminate the disaster risk. Future work could attempt to model these complexities.

Amid the Covid-19 pandemic, countries have increasingly struggled to reach the targets set by the Paris Agreement^2^. As the world nears the point of no return to stop disastrous climate change^45^ and needs costlier investments in high-risk, high-return investments^46^, harnessing global support for climate change mitigation efforts will be an increasingly pressing challenge. Communicating to the public in developed countries the relationship between their present wealth and the carbon emissions of previous generations may increase the support for more ambitious mitigation efforts in these countries.

## Methods

All experiments were conducted online using Qualtrics. We ran the main experiment in November 2020. We ran the additional baseline in June 2022. The experiments were approved by the Institutional Review Board at the National University of Singapore (NUS) (reference number: S-20-040). Informed consent was obtained from all participants. All participants were NUS students, recruited from the pool of the NUS Centre for Behavioural Economics using ORSEE^47^. The earnings were denominated in experimental dollars, converted at the rate: 100 experimental dollars = 1 SGD (at the time of the experiment, the exchange rate was 1 SGD = 0.75 USD). First, we recruited 103 participants for the first generation. The average earnings of the first-generation participants were 4.9 SGD (including the 3 SGD show-up fee) and the median duration of the experiment was 17 min. We used the first-generation data to calculate the outcomes for the second generation. For the second generation, we recruited 103 participants for the Baseline treatment and 101 participants for the History treatment. Each participant in the second generation was assigned a fixed role (either A2 or B2) and matched with four different first-generation pairs. For each pair, the participants made decisions both as a proposer and as a responder. Once we collected all second-generation data, we randomly selected one of the four outcomes for each participant, randomly matched the participant with someone who made choices in the complementary role, randomly selected which participant was the proposer, and calculated the final earnings. If no agreement was reached, we performed a lottery to determine whether the climate disaster occurred or not. The average earnings of the second-generation participants were 7.1 SGD (including the 3 SGD show-up fee) and the median duration of the experiment was 18 min.

Before starting the experiment, participants read the instructions and answered a comprehension quiz. Participants passed the comprehension quiz if they answered at least 5 out of 6 questions correctly. All participants passed the comprehension quiz. After the game, participants answered questions about their attitudes toward climate change^48^ and demographic items (see section S4 of the Supplementary Information for the full text of the instructions, comprehension quiz, and demographic items). The descriptive statistics for the questionnaire are provided in Supplementary Table A6.

In the experiment, the climate change mitigation costs were increasing in the production by the first generation: if we denote the piece rates of A1 and B1 by ${p}_{A}$ and ${p}_{B}$ and the number of completed tasks by *t*_*A*_ and *t*_*B*_, the total climate change mitigation costs were calculated as $C\left({p}_{A},\, {p}_{B},\, {t}_{A},\, {t}_{B}\right)=\tfrac{{\left({p}_{A}{t}_{A}\right)}^{2}}{160}$ + $\tfrac{{\left({p}_{B}{t}_{B}\right)}^{2}}{160}$. The set of piece rates was either {${{p}_{A}}=8,\, {p}_{B}=8$}, {${p}_{A}=10,\, {p}_{B}=6$} or {${p}_{A}=12,\, {p}_{B}=4$}, chosen with equal probabilities and revealed to the participants at the start of the experiment. Environmental costs were convex in production because of the empirical evidence that the relationship between increases in global temperature and carbon emission is non-linear^23,24^. Convexity also ensures that pro-social participants would produce an amount between 0 and 40, thereby increasing the variance of first-stage outcomes needed to identify how the second generation responds to different levels of historical emissions. The scaling parameter was set such that the socially efficient production would not exceed 40 (the maximum number of tasks that participants could complete) for any of the possible piece rates.

To clarify the incentives for the first-generation participants, we explained the relationship between production, earnings, and climate mitigation costs using a table and a figure. We also gave real-time feedback: after completing each task, participants saw their accumulated earnings and generated costs, as well as the subsequent change in these variables from completing one more task. In addition, we framed the game using concrete terms, rather than using abstract language: participants were told that completing each task will build a factory, which will produce a certain number of cars; cars generate earnings but also contribute to climate change, which will need to be mitigated by the next generation (see full instructions in section S4 of the Supplementary Information).

In the History treatment, second-generation participants knew that their endowments and climate mitigation costs were determined by the previous generation; they were also fully informed about the incentives that the first generation faced and the decisions that they made. Participants guessed how much their predecessor produced before the predecessor’s emissions were revealed. Information about beliefs allows us to assess whether the predecessor’s emissions are higher or lower than expected, which might affect behavior in the negotiations stage. Belief elicitation is quite standard in economic experiments and elicitation typically does not affect subsequent decisions^49^. In the Baseline treatment, second-generation participants were informed about the endowments and climate mitigation costs but did not know how these values were determined. In each treatment, participants’ starting endowment was equal to the wealth of their predecessor, plus $600. All second-generation participants were assigned to four pairs of first-generation participants and made decisions for each potential predecessor. Participants had to choose how to divide the climate change mitigation costs using an ultimatum game with a strategy method. First, each participant acted as a proposer and chose how to split the costs between themselves and the other participant. Then, a participant acted as a responder and chose the maximum amount they were willing to pay to accept the proposer’s offer. Both proposers and responders made their decisions using a slider on their screen and saw real-time information about the resulting division of climate mitigation costs and earnings. Initially, the slider was set to $0 (i.e., the proposer would be asking the responder to pay nothing, and the responder would not be willing to pay anything), but participants could not proceed to the next stage until they move or click on the slider. The proposer’s offer was implemented if it was acceptable to the responder; otherwise, it was rejected. If the offer was rejected, there was a 90% chance that participants will earn nothing because of a climate disaster and a 10% chance that the participants would keep their original endowments. Whether the participant was a proposer or a responder was determined by a random draw once all data had been collected.

In our design, participants could either succeed in mitigating climate change and avoid the disaster or fail to reach an agreement and risk disastrous consequences. We made this simplifying assumption to keep our design similar to the collective-risk social dilemma and ultimatum games used in the previous literature^11,37,50^ and to make the game sufficiently simple to understand and analyze. Our design models climate negotiations in which countries need to agree on how to limit the rising temperature beyond some critical level; otherwise, no deal is made. However, in practice, countries could reach a partial agreement, which would reduce the risk of disasters but would not eliminate it. Future research could explore negotiations in which failing to cover all of the mitigation costs would reduce the risk proportionally to the amount paid by the participants.

The inheritance of wealth by the second generation simulates how the development of a country generates wealth for future generations but can also create problems that future generations will need to solve. We added $600 to the endowments in the second generation to ensure that participants were not budget-constrained and could cover the climate mitigation costs. The highest possible value of costs equals $1600 and occurs if participants in the first generation complete all the tasks and productivities are (12, 4). In that case, the endowments of the second-generation participants would be $1080 and $760. Therefore, participants always have enough to jointly cover the costs, and the poorer participant has enough to cover almost half of the costs by themselves.

The destruction of endowments was probabilistic to reflect the probabilistic nature of climate change and in line with the previous literature^8,11,12^. The destruction probability was set to 90%, in line with previous literature^40,50,51^, and ensures that reaching a deal is always socially optimal. After the game, participants provided open-ended comments about how they made their decisions. A quantitative analysis of participants’ open-ended comments is available in section S5 of the Supplementary Information.

The experimental data was analyzed using Stata 14.

### Reporting summary

Further information on research design is available in the Nature Portfolio Reporting Summary linked to this article.

## Supplementary information

Supplementary Information

Reporting Summary

## Data Availability

Experimental data that support the findings of this study have been deposited in OSF: https://osf.io/5rupw/?view_only=bc4ea055ed8045c896ee9e7b34f57a3c.

## References

[1] Jacquet J (2013). Intra-and intergenerational discounting in the climate game. Nat. Clim. Change.

[2] Liu PR, Raftery AE (2021). Country-based rate of emissions reductions should increase by 80% beyond nationally determined contributions to meet the 2 C target. Commun. Earth Environ..

[3] Roelfsema M (2020). Taking stock of national climate policies to evaluate implementation of the Paris Agreement. Nat. Commun..

[4] Uddin M (2017). Climate change and global environmental politics: North-South divide. Environ Policy Law.

[5] Ikeme J (2003). Equity, environmental justice and sustainability: incomplete approaches in climate change politics. Glob. Environ. Change.

[6] Ellermann, C., Höhne, N. & Müller, B. Differentiating historical responsibilities for climate change. In *China’s Responsibility for Climate Change: Ethics, Fairness and Environmental Policy* 71–98 (Policy Press, 2011).

[7] Otto FE, Skeie RB, Fuglestvedt JS, Berntsen T, Allen MR (2017). Assigning historic responsibility for extreme weather events. Nat. Clim. Change.

[8] Anderson B, Bernauer T, Balietti S (2017). Effects of fairness principles on willingness to pay for climate change mitigation. Clim. Change.

[9] Dezső L, Loewenstein G, Steinhart J, Neszveda G, Szászi B (2015). The pernicious role of asymmetric history in negotiations. J. Econ. Behav. Organ..

[10] Dezső L, Loewenstein G (2019). Self-serving invocations of shared and asymmetric history in negotiations. Eur. Econ. Rev..

[11] Gampfer R (2014). Do individuals care about fairness in burden sharing for climate change mitigation? Evidence from a lab experiment. Clim. Change.

[12] Kline R, Seltzer N, Lukinova E, Bynum A (2018). Differentiated responsibilities and prosocial behaviour in climate change mitigation. Nat. Hum. Behav..

[13] Gardiner SM (2004). Ethics and global climate change. Ethics.

[14] Neumayer E (2000). In defence of historical accountability for greenhouse gas emissions. Ecol. Econ..

[15] Shue H (1999). Global environment and international inequality. Int. Aff..

[16] Dellink R (2009). Sharing the burden of financing adaptation to climate change. Glob. Environ. Change.

[17] Müller B, Höhne N, Ellermann C (2009). Differentiating (historic) responsibilities for climate change. Clim. Policy.

[18] Schüssler R (2011). Climate justice: a question of historic responsibility?. J. Glob. Ethics.

[19] Caney S (2005). Cosmopolitan justice, responsibility, and global climate change. Leiden. J. Int. Law.

[20] Den Elzen M, Schaeffer M (2002). Responsibility for past and future global warming: uncertainties in attributing anthropogenic climate change. Clim. Change.

[21] Moellendorf D (2012). Climate change and global justice. Wiley Interdiscip. Rev. Clim. Change.

[22] Friman M, Strandberg G (2014). Historical responsibility for climate change: science and the science–policy interface. Wiley Interdiscip. Rev. Clim. Change.

[23] Armour KC (2016). Climate sensitivity on the rise. Nat. Clim. Change.

[24] Friedrich T, Timmermann A, Tigchelaar M, Timm OE, Ganopolski A (2016). Nonlinear climate sensitivity and its implications for future greenhouse warming. Sci. Adv..

[25] Ferguson MA, Branscombe NR (2010). Collective guilt mediates the effect of beliefs about global warming on willingness to engage in mitigation behavior. J. Environ. Psychol..

[26] Baumeister RF, Stillwell AM, Heatherton TF (1994). Guilt: an interpersonal approach. Psychol. Bull..

[27] Barclay P, Stoller B (2014). Local competition sparks concerns for fairness in the ultimatum game. Biol. Lett..

[28] Kagel JH, Kim C, Moser D (1996). Fairness in ultimatum games with asymmetric information and asymmetric payoffs. Games Econ. Behav..

[29] Ruffle BJ (1998). More is better, but fair is fair: Tipping in dictator and ultimatum games. Games Econ. Behav..

[30] Bilali R, Iqbal Y, Erisen C (2019). The role of lay beliefs about group transgressions in acceptance of responsibility for ingroup harm‐doing. Eur. J. Soc. Psychol..

[31] Čehajić S, Brown R, Gonzalez R (2009). What do I Care? Perceived Ingroup Responsibility and Dehumanization as Predictors of Empathy Felt for the Victim Group. Group Process. Intergroup Relat..

[32] Schelling, T. C. *The Strategy of Conflict* (Harvard University Press, 1960).

[33] Janssen MC (2006). On the strategic use of focal points in bargaining situations. J. Econ. Psychol..

[34] Tversky A, Kahneman D (1974). Judgment under Uncertainty: Heuristics and Biases: Biases in judgments reveal some heuristics of thinking under uncertainty. Science.

[35] Burton-Chellew MN, May RM, West SA (2013). Combined inequality in wealth and risk leads to disaster in the climate change game. Clim. Change.

[36] Gosnell G, Tavoni A (2017). A bargaining experiment on heterogeneity and side deals in climate negotiations. Clim. Change.

[37] Tavoni A, Dannenberg A, Kallis G, Löschel A (2011). Inequality, communication, and the avoidance of disastrous climate change in a public goods game. Proc. Natl Acad. Sci. USA.

[38] Vicens J (2018). Resource heterogeneity leads to unjust effort distribution in climate change mitigation. PLoS ONE.

[39] Mahajan A, Kline R, Tingley D (2022). Collective risk and distributional equity in climate change bargaining. J. Confl. Resolut..

[40] Del Ponte A, Delton AW, Kline R, Seltzer NA (2017). Passing it along: experiments on creating the negative externalities of climate change. J. Polit..

[41] Darley JM, Latané B (1968). Bystander intervention in emergencies: diffusion of responsibility. J. Pers. Soc. Psychol..

[42] Thomas KA, De Freitas J, DeScioli P, Pinker S (2016). Recursive mentalizing and common knowledge in the bystander effect. J. Exp. Psychol. Gen..

[43] Huddy, L. & Del Ponte, A. National identity, pride, and chauvinism–their origins and consequences for globalization attitudes. In *Liberal Nationalism and Its Critics* (Oxford University Press, 2019).

[44] Huddy L, Del Ponte A, Davies C (2021). Nationalism, patriotism, and support for the European Union. Polit. Psychol..

[45] Aengenheyster M, Feng QY, van der Ploeg F, Dijkstra HA (2018). The point of no return for climate action: effects of climate uncertainty and risk tolerance. Earth Syst. Dyn..

[46] Andrews TM, Delton AW, Kline R (2018). High-risk high-reward investments to mitigate climate change. Nat. Clim. Change.

[47] Greiner B (2015). Subject pool recruitment procedures: organizing experiments with ORSEE. J. Econ. Sci. Assoc..

[48] Dijkstra E, Goedhart M (2012). Development and validation of the ACSI: measuring students’ science attitudes, pro-environmental behaviour, climate change attitudes and knowledge. Environ. Educ. Res..

[49] Schotter A, Trevino I (2014). Belief elicitation in the laboratory. Annu Rev. Econ..

[50] Milinski M, Sommerfeld RD, Krambeck H-J, Reed FA, Marotzke J (2008). The collective-risk social dilemma and the prevention of simulated dangerous climate change. Proc. Natl Acad. Sci. USA.

[51] Marotzke J, Semmann D, Milinski M (2020). The economic interaction between climate change mitigation, climate migration and poverty. Nat. Clim. Change.

