# Supplementary material for: Information about historical emissions drives the division of climate change mitigation costs
Source: Nat Commun. 2023 Mar 14;14:1408. doi: 10.1038/s41467-023-37130-7 (PMC10012302; doi:10.1038/s41467-023-37130-7)
Supplement: Supplementary file 1 — Supplementary Information [file 41467_2023_37130_MOESM1_ESM.pdf]

Supplementary Information

S1. Supplementary Tables

Table A1. GLS regression of the share of mitigation costs that proposers offered to cover.

	(1)	(2)	(3)	(4)	(5)	(6)
	H	B	H and B	H	B	H and B
Historical emissions	0.55***	0.12***	0.12**	0.71***	-0.087	-0.079
	(12.79)	(3.55)	(3.17)	(7.08)	(-0.70)	(-0.63)
Climate costs	-0.027	-0.0095	-0.019	-0.025	-0.012	-0.019
	(-1.64)	(-0.56)	(-1.63)	(-1.58)	(-0.69)	(-1.69)
Round	0.0015	0.0023	0.0021	0.00075	0.0013	0.0012
	(0.30)	(0.56)	(0.66)	(0.16)	(0.28)	(0.36)
History treatment			-0.21***			1.02*
			(-6.61)			(2.45)
History × Emissions			0.42***			0.78***
			(7.28)			(4.89)
Relative wealth				-1.21	1.63*	1.57*
				(-1.88)	(2.19)	(2.11)
History × Relative wealth						-2.81**
						(-2.86)
Constant	0.23***	0.43***	0.43***	0.76**	-0.28	-0.25
	(8.29)	(16.19)	(17.96)	(2.77)	(-0.92)	(-0.81)
Observations	404	412	816	404	412	816
R^2	0.45	0.10	0.36	0.46	0.14	0.38

Note: GLS regression with a random effect for each participant, with standard errors clustered at the individual level. *z* statistics in parentheses. Costs are measured in thousands.

* $p < .05$, ** $p < .01$, *** $p < .001$

We include all decisions in the role of a proposer from all participants (four decisions per participant). Historical emissions are calculated as a ratio of climate mitigation costs created by the predecessor to the total climate mitigation costs created by the first generation. Climate costs indicate the total climate mitigation costs to be divided between the proposer and responder (in thousands of experimental dollars). Round indicates the round number (from 1 to 4). History × Emissions is the interaction between historical emissions and the History treatment variable. Relative wealth is calculated as the proposer's endowment, divided by the sum of endowments received by the proposer and the responder. History × Relative wealth is the interaction between relative wealth and the History treatment variable. H denotes the History treatment; B denotes the Baseline. No adjustments were made for multiple comparisons.

Table A2. GLS regression of the maximum share of climate mitigation costs that the responders were willing to cover.

	(1)<br>H	(2)<br>B	(3)<br>H and B	(4)<br>H	(5)<br>B	(6)<br>H and B
Historical emissions	0.36***	0.14***	0.13***	0.32	0.058	0.059
	(5.72)	(3.56)	(3.32)	(1.92)	(0.46)	(0.47)
Climate costs	-0.18***	-0.24***	-0.20***	-0.18***	-0.24***	-0.21***
	(-6.80)	(-7.96)	(-10.47)	(-6.69)	(-7.94)	(-10.38)
Round	0.0068	0.0036	0.0046	0.0070	0.0031	0.0045
	(0.92)	(0.52)	(0.91)	(0.93)	(0.45)	(0.88)
History treatment			-0.15***			-0.027
			(-3.33)			(-0.05)
History × Emissions			0.23**			0.26
			(3.04)			(1.24)
Relative wealth				0.27	0.65	0.59
				(0.28)	(0.76)	(0.69)
History × Relative wealth						-0.28
						(-0.21)
Constant	0.46***	0.65***	0.63***	0.34	0.37	0.38
	(10.16)	(17.85)	(19.85)	(0.83)	(1.03)	(1.05)
Observations	404	412	816	404	412	816
R^2	0.22	0.20	0.21	0.22	0.20	0.22

Note: GLS regression with a random effect for each participant, with standard errors clustered at the individual level. *z* statistics in parentheses. Costs are measured in thousands.
* $p < .05$, ** $p < .01$, *** $p < .001$
We include all decisions in the role of a responder from all participants (four decisions per participant). Historical emissions are calculated as a ratio of climate mitigation costs created by the predecessor to the total climate mitigation costs created by the first generation. Climate costs indicate the total climate mitigation costs to be divided between the proposer and responder (in thousands of experimental dollars). Round indicates the round number (from 1 to 4). History × Emissions is the interaction between historical emissions and the History treatment variable. Relative wealth is calculated as the responder's endowment, divided by the sum of endowments received by the proposer and the responder. H denotes the History treatment; B denotes the Baseline. No adjustments were made for multiple comparisons.

Table A3. GLS regression of the share of climate mitigation costs that the proposers offered to cover (in models 1, 3, and 5) or the maximum share of climate mitigation costs that the responders were willing to cover (in models 2, 4, and 6).

	(1) Proposer	(2) Responder	(3) High emissions proposer	(4) High emissions responder	(5) Low emissions proposer	(6) Low emissions responder
History treatment	-0.094***	-0.084**	0.11***	-0.0092	-0.093***	-0.091**
	(-4.56)	(-2.61)	(4.95)	(-0.31)	(-4.47)	(-2.76)
Climate costs	-0.021	-0.22***	-0.031	-0.21***	0.0056	-0.18***
	(-1.38)	(-9.88)	(-1.51)	(-7.80)	(0.26)	(-6.01)
Round	0.0046	0.0088	0.0038	0.020**	0.00027	-0.011
	(1.24)	(1.64)	(0.71)	(2.81)	(0.05)	(-1.41)
High emissions	0.070***	0.080***				
	(4.09)	(3.88)				
History × High emissions	0.20***	0.098*				
	(6.89)	(2.57)				
Constant	0.45***	0.65***	0.53***	0.72***	0.45***	0.69***
	(24.71)	(24.19)	(27.62)	(25.31)	(17.64)	(20.56)
Observations	749	749	359	359	390	390
R^2	0.30	0.17	0.10	0.12	0.077	0.10

Note: GLS regression with a random effect for each participant, with standard errors clustered at the individual level. *z* statistics in parentheses. Costs are measured in thousands.

* $p < .05$, ** $p < .01$, *** $p < .001$

We include all decisions in the role of a proposer or responder from all participants (four decisions per participant) from both treatments. High emissions indicate whether a participant's predecessor emitted more than the predecessor of the other participant. Observations from cases when both predecessors emitted the same are excluded (8.2% of observations). Climate costs indicate the total absolute value of climate costs to be divided between the proposer and responder (in thousands of experimental dollars). Round indicates the round number (from 1 to 4). Relative wealth is calculated as the responder's endowment, divided by the sum of endowments received by the proposer and the responder. H denotes the History treatment; B denotes the Baseline. No adjustments were made for multiple comparisons.

Table A4. Classification of proposers and responders based on all their decisions. The interval width is 0.1 in the first table (± 0.05), 0.2 in the second table (± 0.1), and 0.4 in the third table (± 0.2).

	**Regression coefficient**					
Interval = 0.1	< -0.05	0 ± 0.05	$0.05 - 0.5$	$0.5 - 0.95$	1 ± 0.05	≥ 1.05
**Proposers in B**	15%	20%	59%	5%	0%	1%
**Proposers in H**	9%	7%	32%	26%	22%	5%
**Responders in B**	38%	15%	29%	15%	0%	4%
**Responders in H**	27%	8%	25%	17%	17%	7%

	**Regression coefficient**					
Interval = 0.2	< -0.1	0 ± 0.1	$0.1 - 0.5$	$0.5 - 0.9$	1 ± 0.1	≥ 1.1
**Proposers in B**	15%	26%	53%	5%	0%	1%
**Proposers in H**	8%	11%	29%	25%	26%	2%
**Responders in B**	33%	21%	27%	13%	2%	4%
**Responders in H**	25%	11%	24%	15%	22%	4%

	**Regression coefficient**					
Interval = 0.4	< -0.2	0 ± 0.2	$0.2 - 0.5$	$0.5 - 0.8$	1 ± 0.2	≥ 1.2
**Proposers in B**	11%	47%	37%	5%	0%	1%
**Proposers in H**	6%	18%	24%	16%	36%	1%
**Responders in B**	26%	37%	18%	12%	3%	4%
**Responders in H**	23%	16%	21%	9%	30%	2%

Note: We regressed the four decisions of each participant (relative share of costs that the proposers offered to pay or the maximum relative share of costs that the responders were willing to pay) on their historical emissions and classified the participant based on the estimated value of the regression coefficient. H denotes the History treatment; B denotes the Baseline.

Table A5. OLS regression with heteroscedasticity-robust standard errors. The dependent variable is the expected probability to reach an agreement.

	(1)	(2)	(3)
Climate costs	-0.33***	-0.26***	-0.32***
	(-7.97)	(-6.33)	(-7.70)
History treatment	0.012	-0.0043	0.0092
	(0.19)	(-0.09)	(0.18)
Inequality in emissions	-0.35*		
	(-2.12)		
History × (Ineq. in emissions)	-0.084		
	(-0.37)		
Inequality in wealth		-2.44*	
		(-2.59)	
History × (Ineq. in wealth)		-0.11	
		(-0.08)	
$p_A = 10, p_B = 6$			-0.047
			(-0.90)
$p_A = 12, p_B = 4$			-0.12
			(-1.96)
History × ($p_A = 10, p_B = 6$)			-0.073
			(-0.90)
History × ($p_A = 12, p_B = 4$)			0.034
			(0.41)
Constant	0.89***	0.85***	0.85***
	(18.46)	(21.41)	(21.36)
Observations	184	184	184
R^2	0.267	0.279	0.251

Note: *t* statistics in parentheses. * $p < .05$, ** $p < .01$, *** $p < .001$

For each first-generation outcome, we calculated the expected probability to reach an agreement by calculating the average acceptance probability across all potential pairings between proposers and responders assigned to the same outcome. The number of observations therefore corresponds to the number of first-stage outcomes for each of the two player types. Inequality in wealth is calculated as the Gini coefficient of the initial endowments received by the proposer and the responder. Inequality in emissions is calculated as the Gini coefficient of the climate mitigation costs created by the ancestors of the proposer and responder. The base category is a treatment with equal piece rates in the first stage ($p_A = 8, p_B = 8$). A conservative HC3 bias correction method is used to calculate heteroscedasticity-robust standard errors. No adjustments were made for multiple comparisons.

Table A6. Mean responses to the questionnaire. Standard deviations are reported in parentheses.

	(1) Baseline	(2) History	(3) Both
Political orientation (-3 = conservative, +3 = liberal)	0.51	0.53	0.52
	(1.26)	(1.19)	(1.22)
People should care more about climate change	3.50	3.58	3.54
	(0.71)	(0.53)	(0.63)
Climate change should be given top priority	3.02	3.23	3.12
	(0.90)	(0.72)	(0.82)
It is annoying to see people do nothing for climate change problems	2.81	2.99	2.90
	(0.97)	(0.81)	(0.90)
People worry too much about climate change	1.17	1.13	1.15
	(0.93)	(0.84)	(0.89)
The seriousness of climate change has been exaggerated	1.03	0.85	0.94
	(1.07)	(0.85)	(0.97)
Climate change is a threat to the world	3.56	3.59	3.58
	(0.61)	(0.49)	(0.55)
Average support for action against climate change	3.12	3.24	3.18
	(0.66)	(0.48)	(0.58)
Age	22.00	22.03	22.01
	(1.87)	(2.17)	(2.02)
Gender (Female = 1)	0.62	0.69	0.66
	(0.49)	(0.46)	(0.48)
Observations	103	101	204

Table A7. Replication of Table 1, replacing the original Baseline treatment with the Baseline with Predecessors.

	(1)<br>H	(2)<br>BP	(3)<br>H and BP	(4)<br>H and BP
Historical emissions	0.53^{***}	0.22^{***}	0.22^{***}	0.38^{***}
	(13.49)	(9.54)	(9.61)	(5.63)
Climate costs	-0.018	-0.012	-0.015	-0.016
	(-0.78)	(-0.71)	(-1.05)	(-1.19)
H treatment			-0.13^{***}	-0.13^{***}
			(-5.03)	(-5.40)
H # Emissions			0.31^{***}	0.31^{***}
			(6.79)	(7.16)
Relative wealth				-1.14^{**}
				(-2.83)
Constant	0.28^{***}	0.41^{***}	0.41^{***}	0.91^{***}
	(9.92)	(20.19)	(22.93)	(5.31)
Observations	90	85	175	175
R^2	0.758	0.543	0.719	0.734

Note: *t* statistics in parentheses. Costs are measured in thousands. No adjustments were made for multiple comparisons.

* $p < 0.05$, ** $p < 0.01$, *** $p < 0.001$

Table A8. Replication of Table A1, replacing the original Baseline treatment with the Baseline with Predecessors.

	(1)<br>H	(2)<br>BP	(3)<br>H and BP	(4)<br>H	(5)<br>BP	(6)<br>H and BP
Historical emissions	0.55***	0.23***	0.17***	0.71***	0.34**	0.096
	(12.79)	(6.61)	(6.35)	(7.08)	(3.21)	(1.07)
Climate costs	-0.027	-0.0032	-0.014	-0.025	-0.0014	-0.014
	(-1.64)	(-0.23)	(-1.54)	(-1.58)	(-0.11)	(-1.53)
Round	0.0015	-0.00060	0.00095	0.00075	-0.00090	0.00063
	(0.30)	(-0.14)	(0.37)	(0.16)	(-0.21)	(0.25)
H treatment			-0.18***			0.61
			(-6.56)			(1.73)
H # Emissions			0.37***			0.61***
			(7.23)			(4.55)
Relative wealth				-1.21	-0.77	0.56
				(-1.88)	(-1.27)	(1.08)
H # Relative wealth						-1.82*
						(-2.20)
Constant	0.23***	0.38***	0.41***	0.76**	0.71**	0.17
	(8.29)	(15.60)	(23.37)	(2.77)	(2.76)	(0.77)
Overall R-squared	0.45	0.17	0.31	0.46	0.17	0.32
Observations	404	372	1188	404	372	1188

Note: *z* statistics in parentheses. Costs are measured in thousands. No adjustments were made for multiple comparisons.

* $p < 0.05$, ** $p < 0.01$, *** $p < 0.001$

Table A9. Replication of Table A2, replacing the original Baseline treatment with the Baseline with Predecessors.

	(1)<br>H	(2)<br>BP	(3)<br>H and BP	(4)<br>H	(5)<br>BP	(6)<br>H and BP
Historical emissions	0.36***	0.17***	0.15***	0.32	0.34*	0.18
	(5.72)	(3.80)	(4.90)	(1.92)	(2.45)	(1.83)
Climate costs	-0.18***	-0.23***	-0.21***	-0.18***	-0.22***	-0.21***
	(-6.80)	(-7.49)	(-12.99)	(-6.69)	(-7.31)	(-12.80)
Round	0.0068	-0.0027	0.0022	0.0070	-0.0032	0.0023
	(0.92)	(-0.41)	(0.56)	(0.93)	(-0.47)	(0.57)
H treatment			-0.15***			-0.36
			(-3.53)			(-0.75)
H # Emissions			0.21**			0.14
			(3.02)			(0.76)
Relative wealth				0.27	-1.20	-0.20
				(0.28)	(-1.42)	(-0.33)
H # Relative wealth						0.50
						(0.43)
Constant	0.46***	0.65***	0.64***	0.34	1.17***	0.73**
	(10.16)	(14.43)	(24.80)	(0.83)	(3.34)	(2.83)
Overall R-squared	0.22	0.16	0.20	0.22	0.16	0.20
Observations	404	372	1188	404	372	1188

Note: z statistics in parentheses. Costs are measured in thousands. No adjustments were made for multiple comparisons.

* $p < 0.05$, ** $p < 0.01$, *** $p < 0.001$

Table A10. Replication of Table A3, replacing the original Baseline treatment with the Baseline with Predecessors.

	(1) Proposer	(2) Responder	(3) High emissions proposer	(4) High emissions responder	(5) Low emissions proposer	(6) Low emissions responder
History treatment	-0.068***	-0.090**	0.077**	-0.0093	-0.077***	-0.10**
	(-3.31)	(-2.61)	(3.20)	(-0.29)	(-3.89)	(-2.99)
Climate costs	-0.016	-0.21***	-0.047*	-0.21***	0.021	-0.19***
	(-1.19)	(-9.13)	(-2.25)	(-7.67)	(1.17)	(-5.75)
Round	0.0026	0.0039	0.0036	0.013	-0.0037	-0.0081
	(0.68)	(0.74)	(0.64)	(1.91)	(-0.65)	(-1.02)
High emissions	0.12***	0.093***				
	(7.35)	(3.56)				
History × High emissions	0.15***	0.084*				
	(5.17)	(2.02)				
Constant	0.43***	0.67***	0.57***	0.73***	0.44***	0.70***
	(23.86)	(20.22)	(23.91)	(21.72)	(18.48)	(17.36)
R^2	0.30	0.16	0.072	0.100	0.058	0.13
Observations	716	716	348	348	368	368

Note: z statistics in parentheses. Costs are measured in thousands. No adjustments were made for multiple comparisons.

* $p < 0.05$, ** $p < 0.01$, *** $p < 0.001$

Table A11. Replication of Table A4, replacing the original Baseline treatment with the Baseline with Predecessors.

	**Regression coefficient**					
Interval = 0.1	< -0.05	0 ± 0.05	$0.05 - 0.5$	$0.5 - 0.95$	1 ± 0.05	≥ 1.05
**Proposers in BP**	10%	25%	49%	19%	1%	2%
**Proposers in H**	9%	7%	32%	26%	22%	5%
**Responders in BP**	31%	15%	27%	19%	1%	6%
**Responders in H**	27%	8%	25%	17%	17%	7%

	**Regression coefficient**					
Interval = 0.2	< -0.1	0 ± 0.1	$0.1 - 0.5$	$0.5 - 0.9$	1 ± 0.1	≥ 1.1
**Proposers in BP**	6%	37%	41%	12%	2%	2%
**Proposers in H**	8%	11%	29%	25%	26%	2%
**Responders in BP**	28%	20%	25%	17%	3%	6%
**Responders in H**	25%	11%	24%	15%	22%	4%

	**Regression coefficient**					
Interval = 0.4	< -0.2	0 ± 0.2	$0.2 - 0.5$	$0.5 - 0.8$	1 ± 0.2	≥ 1.2
**Proposers in BP**	4%	48%	31%	11%	4%	1%
**Proposers in H**	6%	18%	24%	16%	36%	1%
**Responders in BP**	25%	30%	18%	14%	6%	6%
**Responders in H**	23%	16%	21%	9%	30%	2%

Note: We regressed the four decisions of each participant (relative share of costs that the proposers offered to pay or the maximum relative share of costs that the responders were willing to pay) on their historical emissions and classified the participant based on the estimated value of the regression coefficient. H denotes the History treatment; B denotes the Baseline.

Table A12. Replication of Table A5, replacing the original Baseline treatment with the Baseline with Predecessors. The dependent variable is the expected probability to reach an agreement.

	(1)	(2)	(3)
Climate costs	-0.30***	-0.25***	-0.27***
	(-6.98)	(-5.10)	(-6.28)
History treatment	-0.0016	-0.011	-0.0018
	(-0.02)	(-0.20)	(-0.03)
Inequality in emissions	-0.24		
	(-1.22)		
History × (Ineq. In emissions)	-0.18		
	(-0.70)		
Inequality in wealth		-1.60	
		(-1.37)	
History × (Ineq. In wealth)		-1.01	
		(-0.71)	
$p_A = 10, p_B = 6$			-0.088
			(-1.25)
$p_A = 12, p_B = 4$			0.032
			(0.47)
History × $(p_A = 10, p_B = 6)$			-0.034
			(-0.37)
History × $(p_A = 12, p_B = 4)$			-0.11
			(-1.24)
Constant	0.89***	0.85***	0.83***
	(15.74)	(20.83)	(16.39)
Observations	186	186	186
R^2	0.209	0.215	0.205

Note: *t* statistics in parentheses. Costs are measured in thousands. No adjustments were made for multiple comparisons.

* $p < 0.05$, ** $p < 0.01$, *** $p < 0.001$

Table A13. Replication of Table A1 with the added control variables from the questionnaire.

	(1)<br>H	(2)<br>B	(3)<br>H and B	(4)<br>H	(5)<br>B	(6)<br>H and B
Historical emissions	0.55***	0.13***	0.12**	0.71***	-0.083	-0.078
	(12.84)	(3.71)	(3.21)	(7.07)	(-0.67)	(-0.62)
Climate costs	-0.027	-0.0084	-0.019	-0.025	-0.011	-0.019
	(-1.63)	(-0.50)	(-1.59)	(-1.57)	(-0.63)	(-1.64)
Round	0.0015	0.0023	0.0021	0.00074	0.0012	0.0011
	(0.30)	(0.55)	(0.66)	(0.16)	(0.27)	(0.35)
History treatment			-0.21***			1.02*
			(-6.66)			(2.44)
History # Emissions			0.42***			0.78***
			(7.29)			(4.88)
Relative wealth				-1.22	1.63*	1.57*
				(-1.88)	(2.19)	(2.10)
History # Relative wealth						-2.81**
						(-2.86)
Liberal political orientation	0.015	0.0082	0.011*	0.014	0.0081	0.011*
	(1.51)	(1.56)	(2.09)	(1.48)	(1.56)	(2.07)
Support for climate action	0.0088	-0.027*	-0.015	0.0097	-0.026**	-0.014
	(0.31)	(-2.49)	(-1.28)	(0.34)	(-2.58)	(-1.25)
Age	0.0034	0.0058	0.0049	0.0035	0.0060	0.0050
	(0.49)	(1.51)	(1.22)	(0.51)	(1.57)	(1.26)
Female	0.041	0.0088	0.025	0.043	0.0091	0.026
	(1.40)	(0.56)	(1.62)	(1.45)	(0.61)	(1.70)
Constant	0.093	0.37***	0.35***	0.62*	-0.34	-0.34
	(0.65)	(3.89)	(3.85)	(1.99)	(-1.03)	(-1.04)
R^2	0.47	0.12	0.37	0.47	0.17	0.39
Observations	404	412	816	404	412	816

Note: GLS regression with a random effect for each participant, with standard errors clustered at the individual level. *Z* statistics in parentheses. Costs are measured in thousands. No adjustments were made for multiple comparisons.

* $p < .05$, ** $p < .01$, *** $p < .001$.

Table A14. Replication of Table A2 with the added control variables from the questionnaire.

	(1)<br>H	(2)<br>B	(3)<br>H and B	(4)<br>H	(5)<br>B	(6)<br>H and B
Historical emissions	0.35***	0.14***	0.13**	0.32	0.055	0.057
	(5.65)	(3.45)	(3.25)	(1.91)	(0.43)	(0.45)
Climate costs	-0.18***	-0.24***	-0.21***	-0.18***	-0.24***	-0.21***
	(-6.78)	(-7.92)	(-10.45)	(-6.67)	(-7.90)	(-10.36)
Round	0.0068	0.0036	0.0046	0.0070	0.0032	0.0045
	(0.92)	(0.53)	(0.91)	(0.93)	(0.46)	(0.88)
History treatment			-0.15***			-0.024
			(-3.33)			(-0.04)
History # Emissions			0.23**			0.26
			(3.03)			(1.24)
Relative wealth				0.27	0.66	0.59
				(0.27)	(0.77)	(0.70)
History # Relative wealth						-0.29
						(-0.22)
Liberal political orientation	-0.0022	-0.0096	-0.0059	-0.0021	-0.0096	-0.0059
	(-0.16)	(-0.68)	(-0.61)	(-0.15)	(-0.67)	(-0.60)
Support for climate action	0.016	0.021	0.017	0.015	0.021	0.017
	(0.41)	(0.79)	(0.81)	(0.41)	(0.80)	(0.82)
Age	0.00071	0.0037	0.0020	0.00069	0.0038	0.0020
	(0.08)	(0.45)	(0.34)	(0.08)	(0.45)	(0.33)
Female	-0.0085	-0.013	-0.010	-0.0088	-0.013	-0.010
	(-0.20)	(-0.36)	(-0.37)	(-0.21)	(-0.36)	(-0.38)
Constant	0.40	0.52**	0.54***	0.29	0.23	0.29
	(1.71)	(2.60)	(3.60)	(0.58)	(0.57)	(0.75)
R^2	0.22	0.20	0.22	0.23	0.20	0.22
Observations	404	412	816	404	412	816

Note: GLS regression with a random effect for each participant, with standard errors clustered at the individual level. *Z* statistics in parentheses. Costs are measured in thousands. No adjustments were made for multiple comparisons.
* $p < .05$, ** $p < .01$, *** $p < .001$.

Table A15. Effect of historical emissions on the division of costs, proposer's offer, and responder's maximum willingness to pay in the original Baseline and Baseline with Predecessors.

	(1)<br>Cost division	(2)<br>Proposer's offer	(3)<br>Responder's maximum willingness to pay
Historical emissions	0.13**	0.32***	0.24***
	(2.78)	(9.37)	(6.40)
Climate costs	-0.024	-0.015	-0.21***
	(-1.39)	(-1.53)	(-13.11)
Baseline with Predecessors	-0.048	0.039	0.064
	(-1.41)	(1.55)	(1.69)
Baseline with Predecessors × Historical emissions	0.090<br>(1.72)	-0.088<br>(-1.83)	-0.073<br>(-1.21)
Round		0.00061	0.0020
		(0.22)	(0.49)
Constant	0.47***	0.34***	0.57***
	(12.13)	(16.59)	(20.50)
Observations	172	1188	1188
R^2	0.360	0.2469	0.185

* $p < 0.05$, ** $p < 0.01$, *** $p < 0.001$.

Note: *t* or *z* statistics in parentheses, costs measured in thousands.

Model 1 combines Model 1 from Table 1 and Table A7 and includes an interaction effect between Historical emissions and a treatment dummy that indicates Baseline with Predecessors. The dependent variable in Model 1 is the division of costs. Model 2 combines Model 1 from Table A1 and Table A8. The dependent variable in Model 2 is the proposer's offer. Model 3 combines Model 1 from Table A2 and Table A9. Details of the specifications are provided in Tables 1, A1, and A2. The dependent variable in Model 3 is the responder's maximum willingness to pay. All three models pool data from the Baseline and Baseline with Predecessors treatments. No adjustments were made for multiple comparisons.

S2. Supplementary Figures

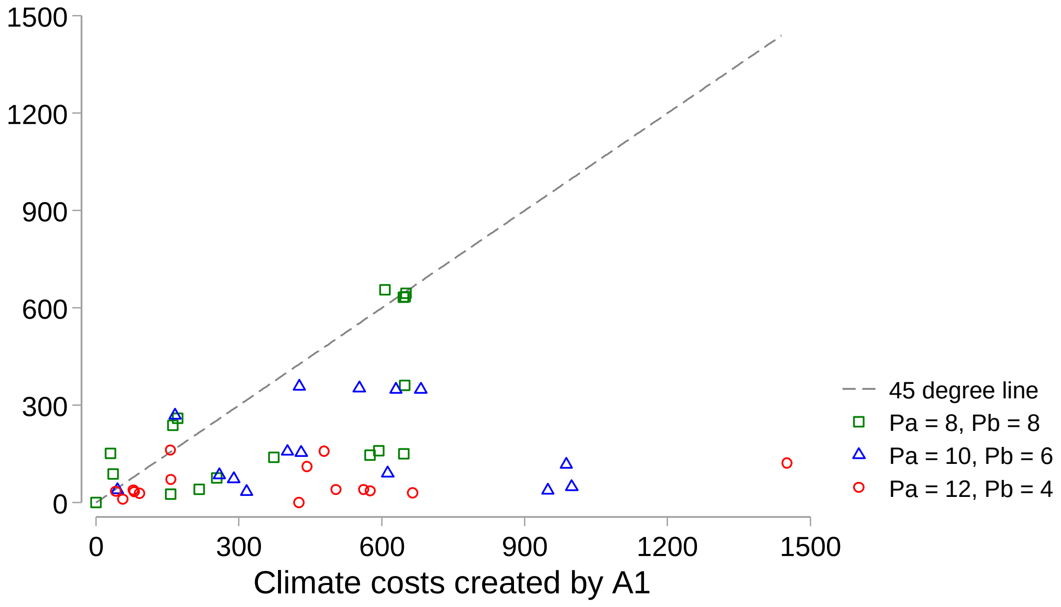

Figure A1. Climate mitigation costs created by each pair of participants in the first generation. Markers indicate the piece rates: equal, moderately unequal, and highly unequal.

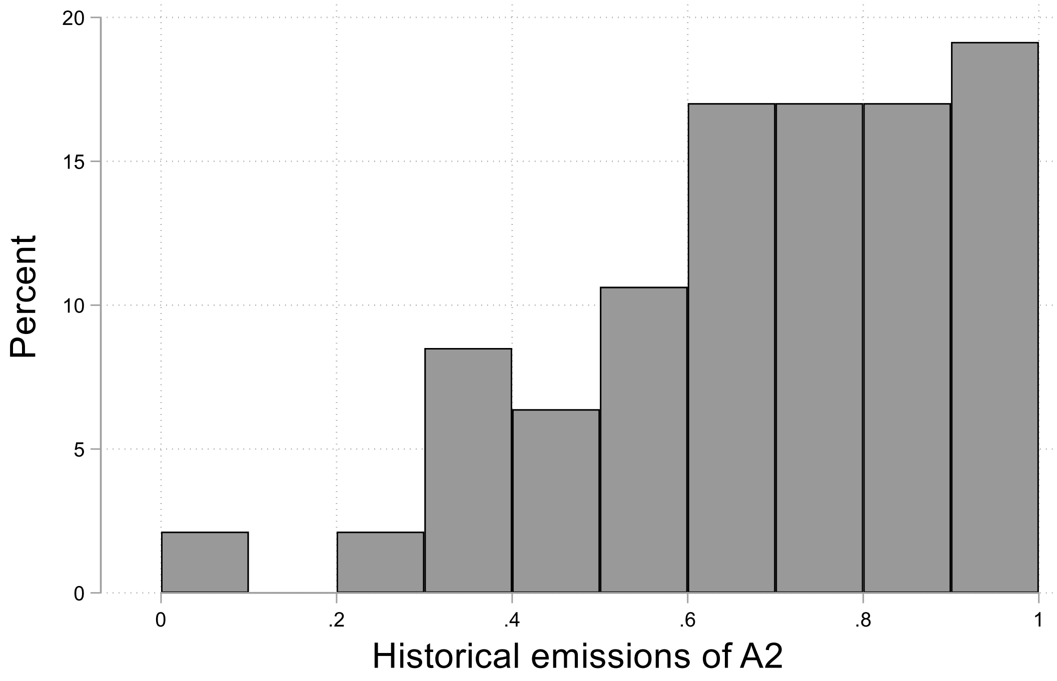

Figure A2. Histogram of historical emissions of A2-type participants in the second generation. The historical emissions of B2-type participants is the opposite, such that the sum should add up to 1.

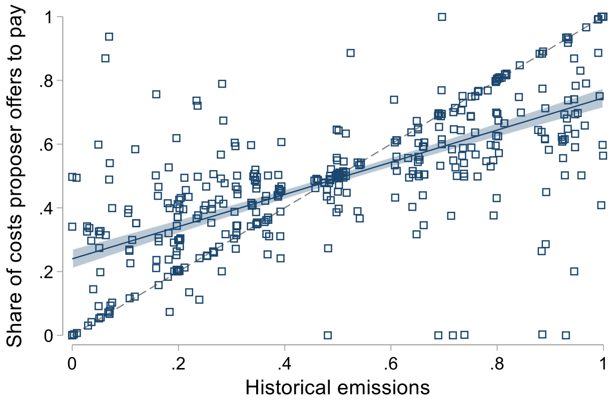

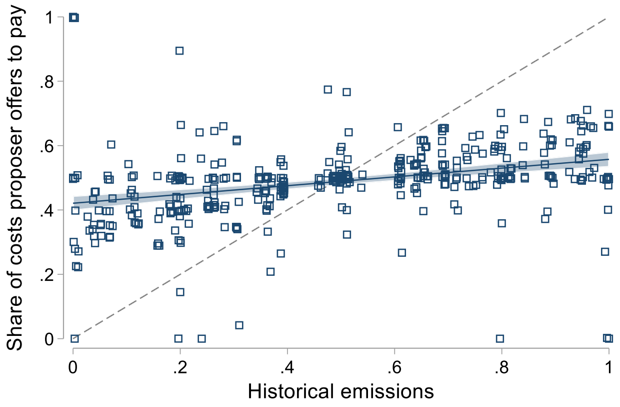

Panel A. Proposer (History treatment)

Panel B. Proposer (Baseline treatment)

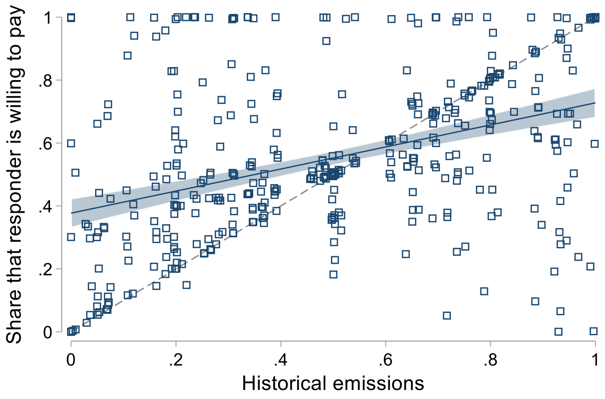

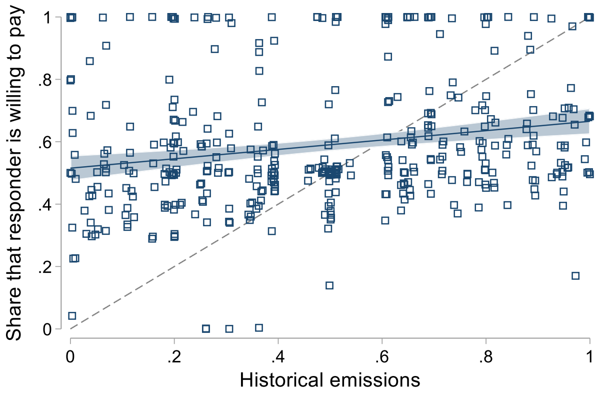

Panel C. Responder (History treatment)

Panel D. Responder (Baseline treatment)

Figure A3. The relationship between the historical emissions and choices of each proposer (top) and responder (bottom). The dashed line marks the 45° line.

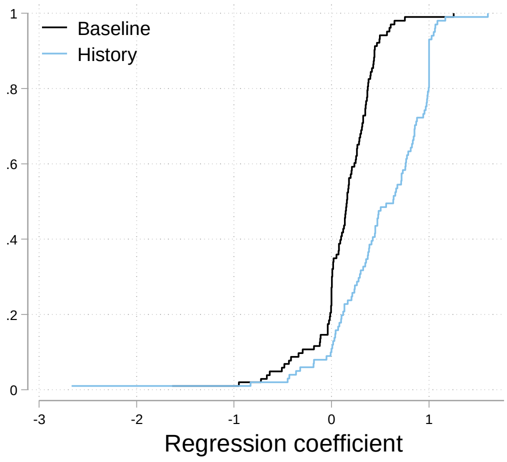

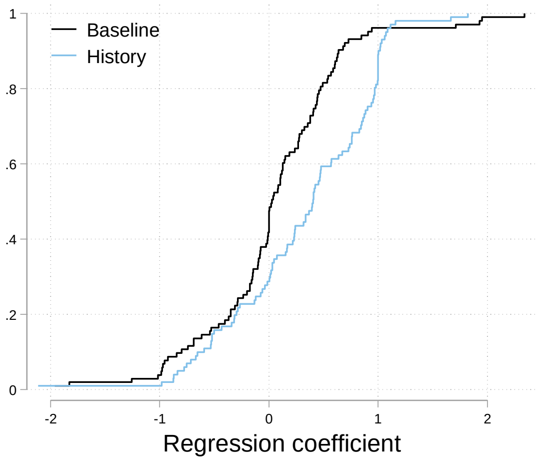

Panel A. Proposers

Panel B. Responders

Figure A4. Cumulative distribution of individually estimated regression coefficients. Coefficients are estimated by regressing the four decisions of each participant (relative share of costs that the proposers offer to pay, or the maximum relative share of costs that the responders are willing to pay) on their historical emissions.

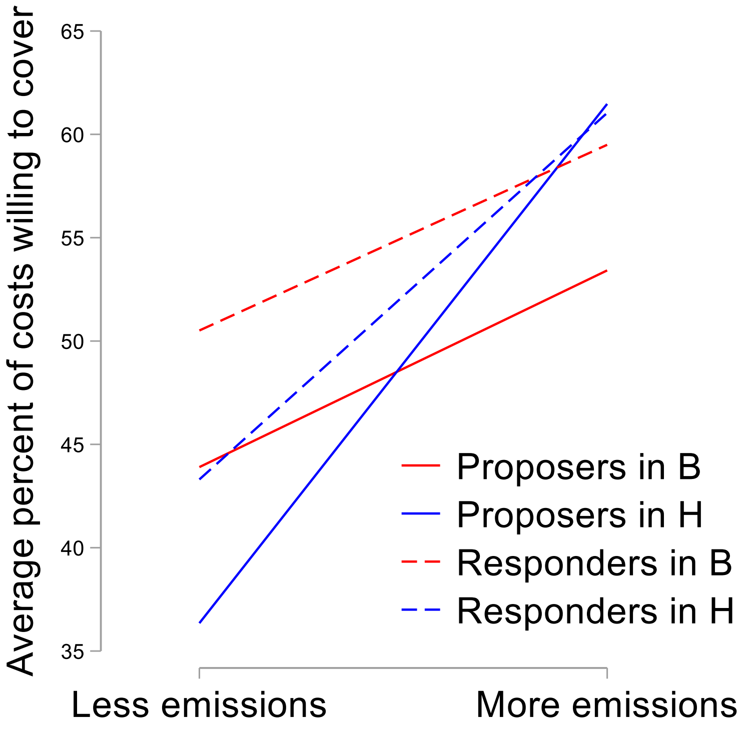

Figure A5. Average percent of climate costs that proposers and responders offer to cover, divided by treatment and historical emissions. **‘**More emissions’ and ‘Less emissions’ refer to whether a participant’s predecessor emitted more or less carbon than the counterpart. Observations from cases when both participants’ predecessors emitted the same are excluded (8.2% of observations). The average percent of costs is calculated by adding the amounts that all participants in that category are willing to cover and dividing it by the total amount of climate mitigation costs. H denotes the History treatment; B denotes the Baseline.

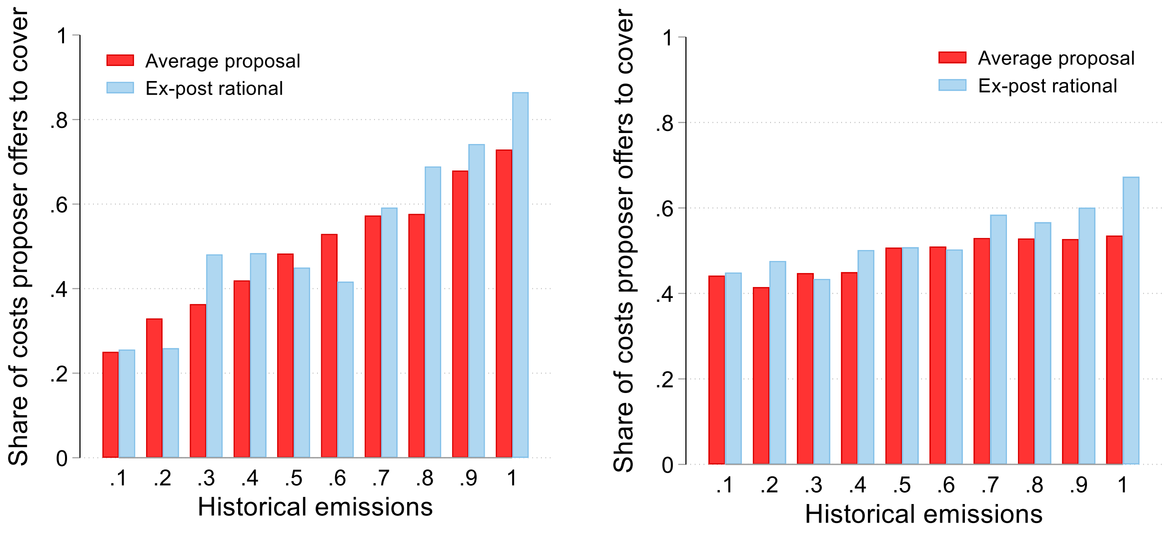

Panel A. History treatment **Panel B**. Baseline treatment

Figure A6. Average offers of the proposers, compared to the ex-post rational offers. Data were binned by aggregating all offers in each range of the proposers' emissions. The ex-post rational offers were calculated by matching each proposer with the responders who have been assigned to the same first-generation outcome and by finding the offer that would have maximized the proposer's expected payoffs.

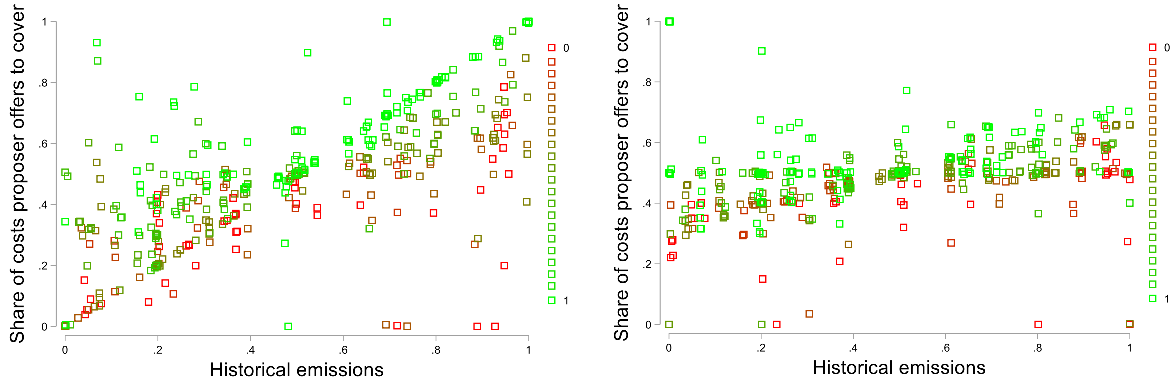

Panel A. History treatment **Panel B**. Baseline treatment

Figure A7. Proposers' offers (dots) and the probability that they will be accepted by responders, from bright red (0% probability) to bright green (100% probability).

S3. Equilibrium predictions

In the first generation, a selfish participant would complete all 40 tasks, as long as the cost of time and effort required to move a slider does not exceed the monetary incentives. Efficiency-oriented participants would complete less than 40 tasks because the climate costs are convex in the number of completed tasks. Specifically, if the cost of effort is zero, participants who seek to maximize total earnings received by all four participants would produce up to the point where the collective marginal benefits (increase in own wealth and the starting endowment of the successor) are equal to the collective marginal costs (increase in the climate change mitigation costs). This holds because in the Nash equilibrium, second-generation participants would always reach an agreement, keeping their endowments and covering the climate mitigation costs. The socially efficient production is summarized in Table A16. Regardless of the productivity parameters, each efficiency-oriented participant would generate $160 of climate change mitigation costs.

Table A16. Socially efficient production in the first generation. The first element indicates productivity or production of A1; the second element indicates it for B1.

Productivities	(8, 8)	(10, 6)	(12, 4)
Equilibrium production	(40, 40)	(40, 40)	(40, 40)
Socially optimal production	(20, 20)	(16, 26.7)	(13.3, 40)

In the second generation, participants play a one-shot ultimatum game using a strategy method. If the responder is not willing to pay the amount offered by the proposer, no agreement is reached and both participants lose all their earnings with probability $p \in (0, 1)$ (in the experiment, we set $p = 0.9$).

Each proposer-responder pair in generation 2 is assigned a set of parameters based on the decisions that another pair of participants made in generation 1. Denote the climate change mitigation costs by C, the endowment of the proposer by E_p and the endowment of the responder by E_r. The proposer makes an offer on how to split C between the two players. The proposal is made by choosing the amount of costs that the proposer is willing to cover; denote this amount by $s_p \in [0, \min\{E_p, C\}]$. The responder chooses the maximum amount of costs they are willing to pay to accept the proposer's offer; denote this amount by $s_r \in [0, \min\{E_r, C\}]$. If the responder is asked to pay less than the maximum willingness to pay that he or she selected, the agreement is reached and the costs are divided according to the proposer's offer (proposer pays s_p and responder pays $C - s_p$); otherwise, no agreement is reached. The expected payoffs of the proposer (π_p) and the responder (π_r) are calculated as:

$$\pi_p = \begin{cases} E_p - s_p, & \text{if } s_p + s_r \geq C \\ (1-p)E_p, & \text{otherwise} \end{cases}$$

$$\pi_r = \begin{cases} E_r - (C - s_p), & \text{if } s_p + s_r \geq C \\ (1-p)E_r, & \text{otherwise} \end{cases}$$

The relevant solution concept in this simultaneous-choice game is the Nash equilibrium. In general, the game has multiple equilibria that also depend on the size of climate change mitigation costs and initial endowments.

If $C > p(E_p + E_r)$, players obtain higher earnings by not reaching an agreement because the mitigation costs exceed the loss from a climate disaster. In that case, there will be no Nash equilibria in which the proposer's offer is accepted because at least one of the players could strictly improve the earnings by reducing the offer or the willingness to pay to zero.

If $C \leq p(E_p + E_r)$, there is a division of costs that ensures that both players prefer reaching an agreement to reaching no agreement. We will specify the range of agreements that could be supported in some Nash equilibrium. The division of costs is determined by the proposer's equilibrium offer s_p^*, generating a payoff of $E_p - s_p^*$ for the proposer and $E_r - (C - s_p^*)$ for the responder if the agreement is reached. First, we will specify the values of s_p^* that cannot occur in any Nash equilibrium, either because one of the players does not have a sufficient endowment or because one of the players would be better off not making an agreement. For the proposer, s_p^* could not be supported in any Nash equilibrium if either of the following conditions hold:

$$s_p^* > E_p$$

$$E_p - s_p^* < (1-p)E_p \;\; \Leftrightarrow \;\; s_p^* > pE_p$$

Since $p \in (0, 1)$, the second condition is stronger than the first one. Therefore, there are no Nash equilibria in which an agreement is reached with $s_p^* > pE_p$ because the proposer could strictly improve the payoffs by choosing $s_p = 0$ and receiving a strictly higher payoff of either $(1-p)E_p$ (if the offer is rejected) or E_p (if the offer is accepted).

Similarly, we can specify the range of agreements that cannot be supported by any Nash equilibrium because the responder would either not have a sufficient endowment or be better off not reaching an agreement. For the responder, s_p^* could not be supported in any Nash equilibrium if either of the following conditions hold:

$$\left(C - s_p^*\right) > E_r \;\; \Leftrightarrow \;\; s_p^* < C - E_r$$

$$E_r - \left(C - s_p^*\right) < (1-p)E_r \;\; \Leftrightarrow \;\; s_p^* < C - pE_r$$

Since $p \in (0, 1)$, the second condition is stronger than the first one. Therefore, there are no Nash equilibria in which an agreement is reached with $s_p^* < C - pE_r$ because the responder could strictly improve the payoffs by choosing $s_r = 0$ and receiving a strictly higher payoff of either $(1-p)E_r$ (if the proposer does not offer to cover all of the costs) or E_r (if the proposer offers to cover all of the costs).

Next, we show that any division of costs $s_p^* \in [C - pE_r, pE_p]$ can be supported in some Nash equilibrium. Specifically, consider equilibria in which the demands of both players add up exactly to C, that is $s_p = s_p^*$ and $s_r = C - s_p^*$. Note that an agreement will be successful because the players cover the entire cost C. This action profile is a Nash equilibrium because neither player could strictly improve their payoffs by unilateral deviation. The equilibrium earnings of the responder are $E_p - s_p^*$; any increase in s_p^* could only lower the earnings and a decrease would lead to no agreement because the responder is choosing $s_r = C - s_p^*$. Payments from no agreement will not exceed $E_p - s_p^*$ because $s_p^* \leq pE_p$. Similarly, the equilibrium earnings of the responder are $E_r - \left(C - s_p^*\right)$ and the responder could not improve

the payoffs by unilateral deviation. Any increase in s_r would generate identical payoffs because the agreement would still be successful, but payoffs would be determined by s_p^*. A decrease in s_r would lead to no agreement, which could not increase the responder's payoffs because $s_p^* \geq C - pE_r$. We conclude that there are Nash equilibria in which the proposer offers to cover any amount $s_p^* \in [C - pE_r, pE_p]$, the responder's chosen maximum willingness to pay is $s_r = C - s_p^*$, and an agreement is reached.

However, there are also Nash equilibria in which no agreement is reached, even if both players would jointly benefit from reaching an agreement. Suppose that neither the proposer nor the responder is willing to cover the entire cost C by themselves, which occurs if $C > pE_p$ and $C > pE_r$. Then there will be an equilibrium in which the proposer offers $s_p = 0$, the responder chooses $s_r = 0$, and no agreement is made. This action profile is a Nash equilibrium because no player could benefit from a unilateral deviation. Specifically, if the other player chooses 0, the only way to reach an agreement is to cover the entire cost C yourself, which will not be profitable if the costs are sufficiently high (i.e., $C > p\max\{E_p, E_r\}$).

We summarize the types of Nash equilibria as follows:

- If $C \in [0, p\max\{E_p, E_r\}]$, the costs are sufficiently small so that at least one player would be better off covering all the costs than reaching no agreement. In that case, an agreement is reached in all the Nash equilibria.
- If $C \in (p\max\{E_p, E_r\}, p(E_p + E_r)]$, neither player is willing to cover the entire cost, but there is a division of costs that makes players at least as well off as when no agreement is made. In that case, there will be Nash equilibria in which an agreement is made and the proposer covers an amount $s_p^* \in [C - pE_r, pE_p]$, but there will also be equilibria in which no agreement is made.
- If $C > p(E_p + E_r)$, there will be no equilibria in which an agreement is made.

In the experiment, we set $p = 0.9$ and the highest possible value of C was set to 1600, which would generate endowments of 760 and 1080, therefore $C < p(E_p + E_r)$. Consequently, in the experiment there always is a Nash equilibrium in which an agreement is reached, although there may also be other equilibria in which no agreement is reached.

S4. Instructions

Instructions for all treatments

Overview

In this study, you can earn money based on your decisions in a game with three other participants. This money is in addition to your $3 payment for completing the survey, which you will earn regardless of what happens in the game.

Your decisions in the game will determine your bonus. For each dollar you have at the end of the game, you will receive 1 cent in real money as an additional bonus. **To receive this bonus and credit for completing the survey, you must complete the game and the questionnaire that follows.**

Please read the instructions carefully as there will be comprehension questions to test your understanding of the instructions. If you do not answer at least 5 out of 6 questions correctly, you will be ineligible to participate in the game and there will not be compensation. You will be given 2 attempts to answer the questions.

After the study, **you will be matched with 3 other participants** and everyone's choices will be carried out to complete the game.

Before proceeding, please read the Participant Information Sheet (PIS) available at the link below:

[Link]

- I have read the PIS and I consent to having my data collected for this study
- I do not consent to having my data collected for this study.

Please enter your NUS Student ID (starts with A). You must enter the same ID you used to register at cbelab.nus.edu.sg.
[textbox]

Payment will be made to the bank account listed in EduRec, which is linked to your student ID. If there are any mistakes in the information provided or if you do not have a bank account registered in EduRec, we will not be able to make the payment.

Participants in Generation 1

Overview of the Experiment

1. **Instructions** of the overall experiment
2. [2 attempts] **6 comprehension questions** - you can proceed only if you get at least 5 out of 6 questions correct
3. **Decision Task** - with short instructions
4. **1 open-ended response question** regarding the decision
5. **Questionnaire** - 10 questions
6. **End**

You can refer to the progress bar below for your progress in the survey.

Please read the instructions carefully as there will be comprehension questions to test your understanding of the instructions. The 'next' button will appear after 60 seconds.

Instructions

In this experiment, you and **three other participants** will be the leaders of two countries at two different stages of economic development. The computer assigned you to be the **leader of Averna (country A) in stage 1** and your teammate will be the leader of A in stage 2. Two other participants will be the leaders of Bessonia (country B) in stages 1 and 2. Your decisions in stage 1 will determine your bonus. In stage 2, your teammate will start with the same amount of money as your bonus in stage 1.

You will choose **how many car factories to build**. To build a factory, you must move a slider on your screen to the “100” position. You can build up to 40 factories, therefore you will see 40 slider tasks on your screen. It is completely up to you how many factories to build: you can exit the stage at any time. The **more factories** you build, the **more cars** you will produce and the **more money** you will make: you will earn $1 per car produced. You will produce **[4/6/8/10/12] cars** per factory and B will produce **[12/10/8/6/4] cars** per factory.

However, **cars generate carbon emissions** that have a 90% chance of causing climate change that would **destroy the earnings of the leaders of A and B in stage 2**. To mitigate climate change, **stage 2 leaders** will have to **divide and pay mitigation costs**. The more cars you and B produce, the exponentially higher the mitigation costs will be. This means that the mitigation costs will increase faster the more cars you produce, as illustrated in the figure below. For example, producing the first 40 cars will increase the mitigation costs by $10. But if you have already produced 400 cars, the next 40 cars will increase the mitigation costs by $210. The total mitigation costs in stage 2 are calculated by adding the costs generated by cars in A to the costs generated by cars in B.

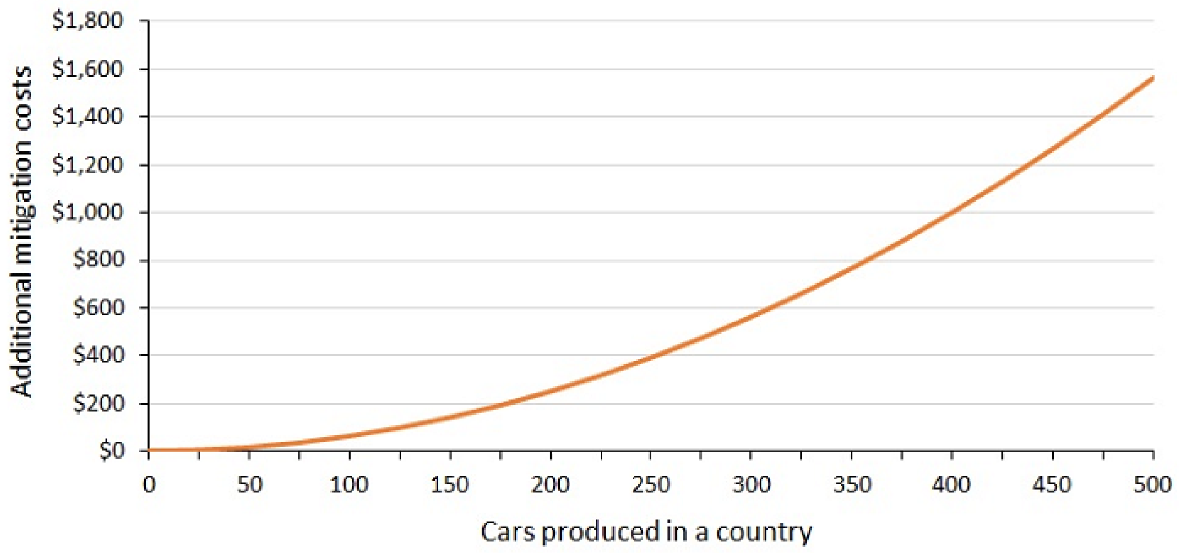

In stage 2, **your teammate** will negotiate with the leader of B how to split these costs. To pay for these costs, **your teammate will receive a new pot of money that is equal to your earnings in stage 1**, plus an additional $600 to cover expenses. Similarly, the new leader of B will receive the same amount of money that the previous leader of B earned in stage 1, plus $600. To conduct the negotiation, each leader will propose how to divide the costs and indicate the maximum they are willing to pay to accept a proposal. After both leaders have submitted their decisions, the computer will select one leader as the proposer, and their proposed division will be carried out if it is compatible with the maximum amount that the other leader is willing to pay. If the negotiation fails, the leaders of A and B in stage 2 will lose all their money with a 90% probability. However, you and the other participant from stage 1 will keep all of your earnings regardless of what happens in stage 2.

Summary:

- **Four participants** take part in this experiment: two are leaders of A and B in stage 1, the other two are leaders of A and B in stage 2. **You are the leader of A in stage 1**.
- **Stage 1** leaders can do tasks to build factories. **Factories** produce **cars**, which increase **earnings** in stage 1 but also increase the **climate change mitigation costs** in stage 2.
- **Stage 2** leaders will have to agree on how to **divide the mitigation costs** or face a 90% risk to lose all their money.
- The **starting balance** of leaders in stage 2 is equal to **their teammate's earnings** in stage 1 + $600.

Quiz for Generation 1 participants *[randomized order of response options; correct answer bolded]*

1. How can you increase your earnings in this game?

    a. By mitigating carbon emissions
    b. By taking earnings from your teammate
    c. By building more factories

d. By negotiating with the leader of B

2. Who will pay the cost of mitigating carbon emissions?
   a. You
   b. Leader of B in stage 1
   c. **Leaders of A and B in stage 2**
   d. The country that produced more emissions

3. What are the roles of the participants in this game?
   a. Two participants are the leaders of A and B in stage 1. Other two participants are the leaders of A and B in stage 2.
   b. One participant is the leader of A, another participant is the leader of B.
   c. One participant is the leader of A in stages 1 and 2. Another participant is the leader of B in stages 1 and 2.
   d. One participant is the leader of A in stage 1. Another participant is the leader of A in stage 2.

4. What will be the starting balance of your teammate?
   a. $600
   b. $600 + the amount you earned
   c. $600 + the climate change mitigation costs created in your country
   d. $600 + number of car factories your teammate built

5. What is the maximum number of factories that you can build?

   a. 10
   b. 20
   c. 40
   d. 60

6. What will your teammate in stage 2 have to do?

   a. Propose how to build more factories
   b. Propose how to divide the mitigation costs with the other leader
   c. Propose a strategy for reducing emissions
   d. Propose the maximum number of cars in each country

Factory Decision

[The example shows information seen by participants with $12 piece-rate. Information in the text, table and figure was updated accordingly for participants with other piece-rate values.]

Each factory you build will produce **12 cars** and each factory B builds will produce **4 cars**. Now, you can choose how many factories to build (from 0 to 40). Each built factory will produce **12 cars** and therefore increase your earnings by **$12**. The table and figure below show the relationship between the number of factories you build and the **cars** produced, the **earnings** those cars would generate for you and your teammate and the additional **mitigation costs** that this production would generate in stage 2. For your convenience, the table displays the earnings for every ten factories you build.

As a reminder, your teammate in stage 2 will start off with the amount that you have earned in this stage + $600. However, your teammate will also have to negotiate with the other leader in stage 2 to split the mitigation costs for emissions that stage 1 leaders generated. If they fail to agree on how the costs should be divided, there will be a 90% chance that they will lose all their earnings.

Number of factories you built	0	10	20	30	40
Cars produced	0	120	240	360	480
Your earnings	$0	$120	$240	$360	$480
Additional mitigation costs borne by participants in stage 2	$0	$90	$360	$810	$1440

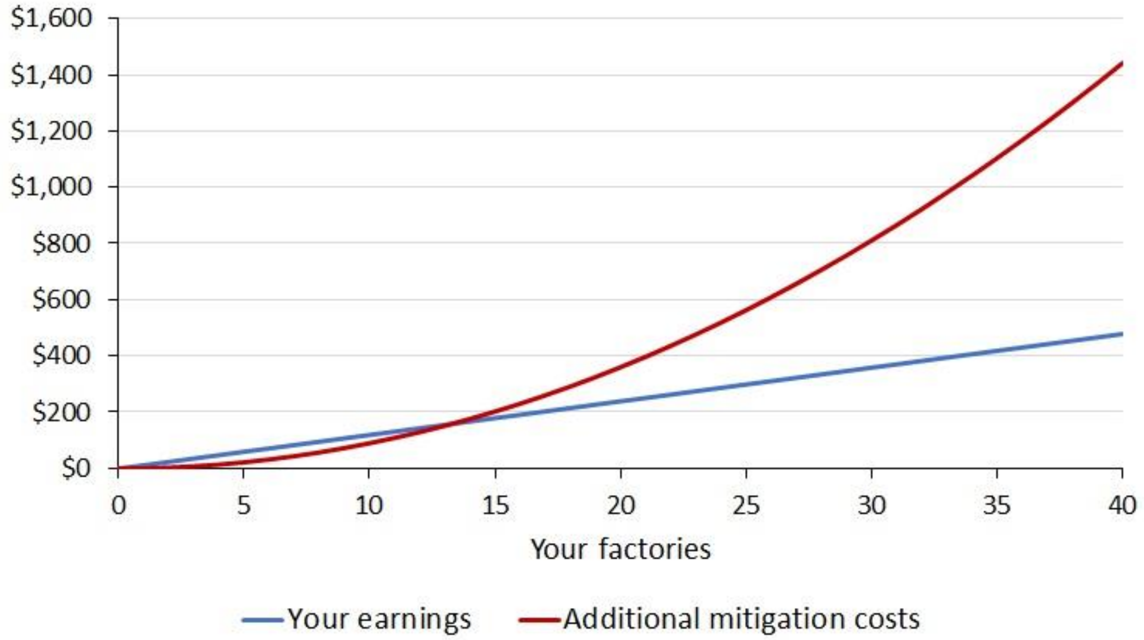

Tasks

Below, you see 40 sliders, which represent the maximum of 40 factories that you could build. **To build a factory, you must move the slider to the "100" position.** Initially, sliders are in random positions between 0 and 200.

As you complete the tasks, you will see **real-time information** about the total **number of factories** built so far, the total number of **cars produced** by those factories, your **earnings** so far, the **current mitigation costs** and the **additional mitigation costs** that building the next factory will generate. There is no time limit for the stage and it is up to you to decide whether to build all the factories, some factories, or none at all. You can exit this stage whenever you want.

[The figure below illustrates the information seen while completing the real-effort tasks; initially, sliders were shuffled and had to be moved to "100" position. A total of 40 sliders

were shown on the screen. Participants saw real-time information about accumulated and marginal earnings and costs, updated after each completed task.]

Factories built	Cars produced	Your earnings	Additional mitigation costs
**2**	**24**	**$24**	**$3.6**

The next factory will increase your earnings by **$12** and increase mitigation costs by **$4.5**

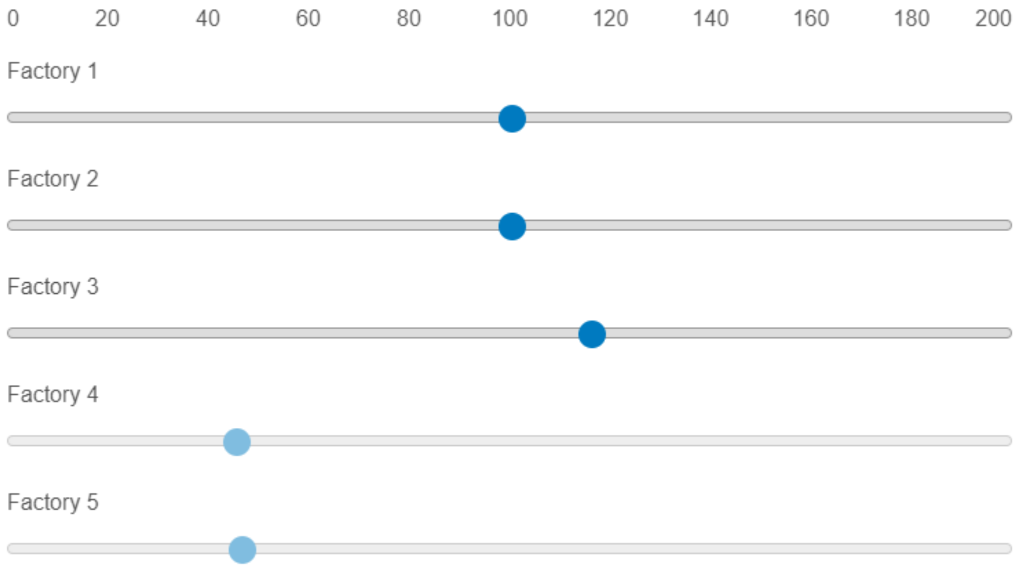

Generation 2 participants in the History treatment

Overview of the Experiment

1. **Instructions** of the overall experiment
2. **6 comprehension questions** - you can proceed only if you get at least 5 questions correct (2 attempts)
3. [4 rounds] **Question** to guess other players' decisions
4. [4 rounds] **Decision Task**
5. **1 open-ended response question** regarding your decision
6. **Questionnaire** - 10 questions
7. **End** of survey

You can refer to the progress bar below for your progress in the survey.

Please read the instructions carefully as there will be comprehension questions to test your understanding of the instructions. The 'next' button will appear after 60 seconds.

Instructions

In this experiment, you and **three other participants** will be the leaders of two countries at two different stages of economic development. Your teammate was the leader of Averna (country A) in stage 1 and **you will be the leader of A in stage 2**. Two other participants are the leaders of Bessonia (country B) in stages 1 and 2. Below, we will describe what choices the two leaders had to make in Stage 1. Please read the explanation closely because later we will ask you to answer a few quiz questions and to guess what the two leaders chose.

Stage 1

In stage 1, each leader was choosing **how many car factories to build** in their country. To build a factory, the participant had to complete a task (move a slider on the computer screen). Each leader could build up to 40 factories. It was completely up to them how many factories to build: they could exit the stage at any time. **Factories produce cars, and each leader earned $1 for each car produced.**

How many cars each factory in A and B produces depends on the resources in A and B. Since A has the same amount or more resources than B, each factory in A will produce either more or the same number of cars as each factory in B. In particular, each of the following three outcomes were equally likely:

1. Each factory in **A** and **B** produced **8 cars**.
2. Each factory in **A** produced **10 cars** whereas each factory in **B** produced **6 cars**.
3. Each factory in **A** produced **12 cars** whereas each factory in **B** produced **4 cars**.

One of these three outcomes was randomly chosen by the computer. The leaders in stage 1 knew how many cars their factories will produce when making their decisions.

However, **cars generate carbon emissions** that have a 90% chance of causing climate change that would **destroy your earnings**. To mitigate climate change, **you and the leader of B** will have to **divide and pay the mitigation costs**. The more cars your teammate and the first leader of B produce, the exponentially higher the mitigation costs will be. The total mitigation costs are calculated by adding the costs generated by cars in A to the costs generated by cars in B in stage 1.

<u>Stage 2</u>

In stage 2, you and the new leader of B will have to pay the costs to mitigate climate change, which is a result of carbon emissions by cars in your countries. The two of you will have to decide which part of the costs will be paid by A and which part by B. If you fail to reach an agreement, there will be a 90% chance that you and the leader of B will lose all the earnings. Note that leaders of stage 1 have already received their earnings, thus whether or not you reach an agreement will not affect them. To pay for these costs, you will receive a pot of money that is equal to **the earnings of your teammate in stage 1**, plus an additional **$600**. Similarly, the new leader of B will receive the same amount of money that the previous leader of B earned in stage 1, plus $600.

The procedure for deciding how to divide the costs is as follows. One of you will act as the **Proposer.** The Proposer will **propose how to divide the costs.** The other participant will act as the **Responder.** The Responder will choose the **maximum amount of money they are willing to pay** to accept the Proposer's offer (e.g. "I would accept an offer if I am asked to pay less than $100").

Each of you will make a decision both as a Proposer and as a Responder. Afterward, the computer will randomly choose who is the Proposer and who is the Responder.

- If the Responder is **willing** to pay the costs asked by the Proposer (i.e. the maximum amount the Responder is willing to pay is **higher** than the amount the Proposer asked), the agreement will be **successful**, and each participant will **pay the costs suggested by the Proposer**.
- If the Responder is **not willing** to pay the costs asked by the Proposer (i.e. the maximum amount the Responder is willing to pay is **lower** than the amount the Proposer asked), the agreement will **not be successful**, and both of you will **lose all of your money with 90% probability**.

Summary:

- **Four participants** take part in this experiment: two are leaders of A and B in stage 1, the other two are leaders of A and B in stage 2. **You are the leader of A in stage 2**.
- **Stage 1** leaders can do tasks to build factories. **Factories** produce **cars**, which increase **earnings** in stage 1 but also increase the **climate change mitigation costs** in stage 2.
- **Stage 2** leaders will have to agree on how to **divide the mitigation costs** or face a 90% risk to lose all their money.
- The **starting balance** of leaders in stage 2 is equal to **their teammate's earnings** in stage 1 + $600.

Quiz for Generation 2 participants [randomized order of response options; correct answer bolded]

1. What will you and the leader of B have to decide on?

    a. How to divide the mitigation costs
    b. How to select the role of the Proposer and Responder

c. How much money to give to the other leader
d. How to increase car emissions

2. What is the role of the Proposer?

a. To propose how to divide the mitigation costs
b. To propose strategies of reducing car emissions
c. To propose the roles of the participants
d. To propose how many cars each leader has

3. What is the role of the Responder?
a. To respond to the Proposer's suggestions of strategies for reducing car emissions
b. To choose the maximum amount they are willing to pay to accept the Proposer's offer
c. To agree with the Proposer's assignment of roles
d. To decide on the minimum number of cars they want to have

4. What happens to you and the leader of B if no agreement is reached?

a. There is a 50% chance that you will lose your money
b. There is a 90% chance that you will lose your money
c. There is a 90% chance that you will keep your money
d. You will lose your money for sure

5. Suppose that the Responder indicates that the maximum amount of money they are willing to pay is $150. Which offer would lead to a successful agreement?
a. Proposer asks the Responder to pay $50
b. Proposer asks the Responder to pay $200
c. Responder asks the Proposer to pay $120
d. None of the above

6. What will be your role in the game?
a. You will be the Proposer
b. You will be the Responder
c. You will be either the Proposer or the Responder
d. You will observe the decisions of a Proposer and a Responder

[Next page – the example is for participants whose predecessors had a $12 piece-rate]
You will play 4 rounds of the game. In each round, you will be matched with one potential teammate (leader of A in stage 1). In each round, you will guess how many factories your teammate built and then make your decision as a Proposer and as a Responder.

One of these 4 rounds will be selected at random, and your choices for that round will determine your earnings. The leader of B in stage 2 will be assigned to the same two leaders of A and B in stage 1 as you. We will calculate your final earnings once we receive the decisions of the leader of B in stage 2.

[Next page]

It is round 1 and you will be matched with potential teammate #1.

Round 1

Each factory in A produced **12 cars** and each factory in B produced **4 cars**.

Next, you will be guessing the number of factories that teammate #1 has built in stage 1.

Your Guess about Teammate's Factory Decision

The table below shows how many **factories** could have been built by your teammate in stage 1, the **cars** that would have been produced, the **earnings** that this production would generate for your teammate and for yourself, and the **additional mitigation costs** that you and the leader of B would have to pay in stage 2. The more cars are produced in a country, the exponentially higher the mitigation costs will be, as shown in the figure below. Your teammate knew all this information. For your convenience, the table displays the earnings for every ten factories.

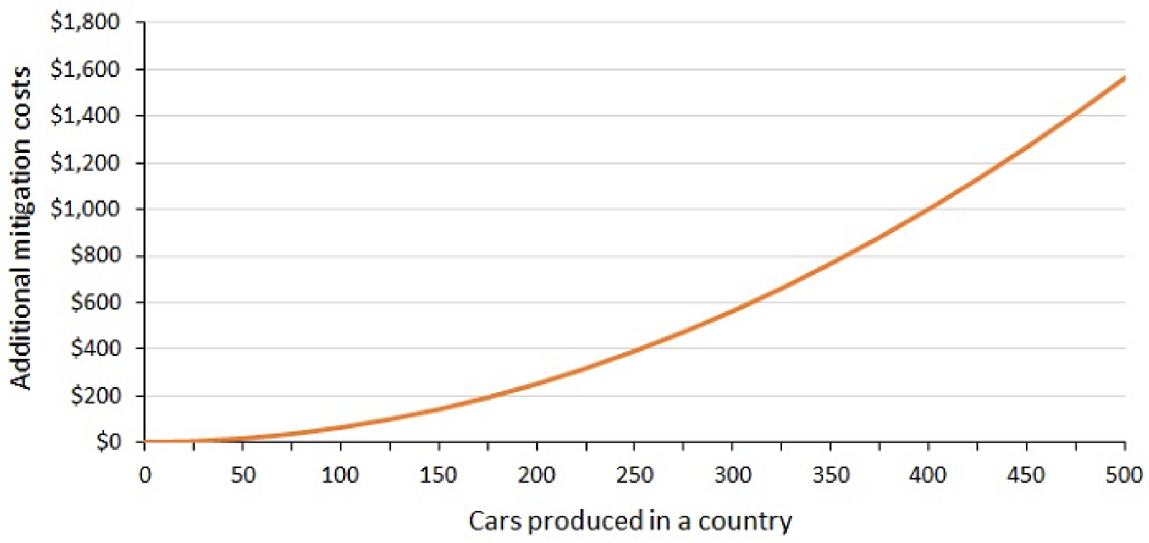

Number of factories in A	0	10	20	30	40
Cars produced	0	120	240	360	480
Earnings of your teammate = Your starting funds in stage 2	$0	$120	$240	$360	$480
Additional mitigation costs borne by you and the second leader of B	$0	$90	$360	$810	$1440

Please guess how many factories your teammate in stage 1 has built. Recall that the maximum number of factories that can be built is 40.

How many factories do you think your teammate has built? [0-40]

[Next page]

Decisions in Stage 1 *[X and Y were replaced by the values depending on the generation 1 decisions]*

The table below summarizes what happened in stage 1. Your teammate built **X out of 40 factories** whereas the leader of B in stage 1 built **Y out of 40** factories. Computer chose that each factory in A produced **12** cars and each factory in B produced **4** cars. It means that A produced **X*12** cars and generated climate mitigation costs equal to **$XX.** B produced **Y*4** cars and generated climate mitigation costs equal to **$YY.** The total mitigation costs that will have to be paid by you and the new leader of B are **$(XX+YY).**

Your teammate earned **$XXX** in stage 1. Thus, you will start stage 2 with the same amount of money that your teammate earned and an additional $600, which adds up to a total of **$(XXX+600)**.

The first leader of B earned **$YYY** in stage 1. Thus, the second leader of B will start stage 2 with the same amount of money that their teammate earned and an additional $600, which adds up to a total of **$(YYY+600).**

[Table summarizes outcomes from generation 1]

	**Factories**	**Cars**	**Generated mitigation costs**	**Earnings**	**Money in stage 2**
**Country A**					
**Country B**					
**Total**					

Now, we will ask you to choose how to divide the **$(XX+YY)** climate change mitigation costs that were generated by A and B in stage 1. These costs are a result of a total of **(X+Y)** car factories that have produced **(X*12+Y*4)** cars in both A and B. To pay for these costs, you will receive **$(XXX+600)** and the leader of B in stage 2 will receive **$(YYY+600)**.

<u>Proposer's decision</u>

If you are chosen to be the Proposer, you will propose how to divide the **$(XX+YY)** mitigation costs. If B rejects your offer, there will be a 90% chance that you will lose all your money. Please choose how much to ask B to pay:

[Slider from $0 to $(XX+YY)]

<u>Responder's decision</u>

If you are chosen to be the Responder, you will decide whether to accept the Proposer's offer. Please choose the highest amount you are willing to pay to accept an offer:

[Slider from $0 to $(XXX+600)]

[Next page – participants make decisions in rounds 2, 3 and 4]

Generation 2 participants in the Baseline treatment

Overview of the Experiment

1. **Instructions** of the overall experiment
2. **6 comprehension questions** - you can proceed only if you get at least 5 questions correct (2 attempts)
3. [4 rounds] **Decision Task**
4. **1 open-ended response question** regarding your decision
5. **Questionnaire** - 10 questions
6. **End** of survey

You can refer to the progress bar below for your progress in the survey.

Please read the instructions carefully as there will be comprehension questions to test your understanding of the instructions. The 'next' button will appear after 60 seconds.

Instructions

In this experiment, you and **another participant** will be the leaders of two countries. You will be the **leader of Averna (country A)** and the other participant will be the leader of Bessonia (Country B).

You and the leader of B will have to pay the costs to mitigate climate change, which is a result of carbon emissions by cars in your countries. The two of you will have to decide which part of the costs will be paid by A and which part by B. If you fail to reach an agreement, there will be a 90% chance that you and the leader of B will lose all the earnings. To pay for these costs, both you and the leader of B will receive a certain amount of money.

The procedure for deciding how to divide the costs is as follows. One of you will act as the **Proposer.** The Proposer will **propose how to divide the costs.** The other participant will act as the **Responder.** The Responder will choose the **maximum amount of money they are willing to pay** to accept the Proposer's offer (e.g. "I would accept an offer if I am asked to pay less than $100").

Each of you will make a decision both as a Proposer and as a Responder. Afterward, the computer will randomly choose who is the Proposer and who is the Responder.

- If the Responder is **willing** to pay the costs asked by the Proposer (i.e. the maximum amount the Responder is willing to pay is **higher** than the amount the Proposer asked), the agreement will be **successful**, and each participant will **pay the costs suggested by the Proposer**.
- If the Responder is **not willing** to pay the costs asked by the Proposer (i.e. the maximum amount the Responder is willing to pay is **lower** than the amount the Proposer asked), the agreement will **not be successful**, and both of you will **lose all of your money with 90% probability**.

Summary:

- **Two participants** take part in this experiment: one is the leader of A and another is the leader of B. **You are the leader of A**.

- The two leaders will have to agree on how to **divide the mitigation costs** or face a 90% risk to lose all their money.

[Next page]
[Quiz, identical to the History treatment]
[Next page]

There are a total of 4 decision rounds, where you will have to make decisions as the proposer and the responder. The climate change mitigation costs and the amount that you will receive will be different in each round.

At the end of the survey, the computer will randomly select one of the four rounds and your earnings will be based on that decision round. Your final earnings will be calculated after the leader of B has made their decisions for that decision round.

[Next page]

Round 1

In round 1, we will ask you to choose how to divide the **$(XX+YY)** climate change mitigation costs. These costs are a result of a total of **(X+Y)** car factories that have produced **(X*12+Y*4)** cars in both A and B. To pay for these costs, you will receive **$(XXX+600)** and the leader of B in stage 2 will receive **$(YYY+600)**.

<u>**Proposer's decision**</u>
If you are chosen to be the Proposer, you will propose how to divide the **$(XX+YY)** mitigation costs. If B rejects your offer, there will be a 90% chance that you will lose all your money. Please choose how much to ask B to pay:

[Slider from $0 to $(XX+YY)]

<u>**Responder's decision**</u>
If you are chosen to be the Responder, you will decide whether to accept the Proposer's offer. Please choose the highest amount you are willing to pay to accept an offer:

[Slider from $0 to $(XXX+600)]

[Next page – participants make decisions in rounds 2, 3 and 4]

Generation 2 participants in the Baseline with Predecessors treatment

Overview of the Experiment

1. **Instructions** of the overall experiment
2. **6 comprehension questions** - you can proceed only if you get at least 5 questions correct (2 attempts)
3. [4 rounds] **Decision Task**
4. **1 open-ended response question** regarding your decision
5. **Questionnaire** - 10 questions
6. **End** of survey

You can refer to the progress bar below for your progress in the survey.

Please read the instructions carefully as there will be comprehension questions to test your understanding of the instructions. The 'next' button will appear after 60 seconds.

Instructions

In this experiment, you and **another participant** will be the leaders of two countries. You will be the **leader of Averna (country A)** and the other participant will be the leader of Bessonia (Country B).

You and the leader of B will have to pay the costs to mitigate climate change, which is a result of carbon emissions by cars in your countries. The two of you will have to decide which part of the costs will be paid by A and which part by B. If you fail to reach an agreement, there will be a 90% chance that you and the leader of B will lose all the earnings. To pay for these costs, both you and the leader of B will receive a certain amount of money.

The money you receive to pay for these costs is determined by the decisions that two other participants made in a different game. One of these participants was the leader of country A and another was the leader of country B. These two participants made decisions in a different type of experiment and received earnings for it. The amount that you and the current leader of B will have to pay to mitigate climate change is also a result of the decisions of the two previous leaders of A and B.

The procedure for deciding how to divide the costs is as follows. One of you will act as the **Proposer.** The Proposer will **propose how to divide the costs.** The other participant will act as the **Responder.** The Responder will choose the **maximum amount of money they are willing to pay** to accept the Proposer's offer (e.g. "I would accept an offer if I am asked to pay less than $100").

Each of you will make a decision both as a Proposer and as a Responder. Afterward, the computer will randomly choose who is the Proposer and who is the Responder.

- If the Responder is **willing** to pay the costs asked by the Proposer (i.e. the maximum amount the Responder is willing to pay is **higher** than the amount the Proposer asked), the agreement will be **successful**, and each participant will **pay the costs suggested by the Proposer**.
- If the Responder is **not willing** to pay the costs asked by the Proposer (i.e. the maximum amount the Responder is willing to pay is **lower** than the amount the Proposer asked), the agreement will **not be successful**, and both of you will **lose all of your money with 90% probability**.

Summary:

- **Two participants** take part in this experiment: one is the leader of A and another is the leader of B. **You are the leader of A**.
- The two leaders will have to agree on how to **divide the mitigation costs** or face a 90% risk to lose all their money.

[Next page]
[Quiz, identical to the Baseline treatment]
[Next page]

There are a total of 4 decision rounds, where you will have to make decisions as the proposer and the responder. In each round, you will be matched with a different pair of previous leaders of A and B. The climate change mitigation costs and the amount that you will receive will depend on the decisions that the previous leaders made and therefore they will change in each round. The current leader of B will be assigned to the same two previous leaders of A and B as you.

At the end of the survey, the computer will randomly select one of the four rounds and your earnings will be based on that decision round. Your final earnings will be calculated after the leader of B has made their decisions for that decision round.

[Next page]

Round 1

In round 1, we will ask you to choose how to divide the **$(XX+YY)** climate change mitigation costs. These costs have been determined by the decisions that the previous leaders of A and B made and are a result of a total of **(X+Y)** car factories that have produced **(X*12+Y*4)** cars in both A and B. To pay for these costs, you will receive **$(XXX+600)** and the leader of B in stage 2 will receive **$(YYY+600)**. These values have also been determined by the decisions that the previous leaders of A and B made.

<u>Proposer's decision</u>
If you are chosen to be the Proposer, you will propose how to divide the **$(XX+YY)** mitigation costs. If B rejects your offer, there will be a 90% chance that you will lose all your money. Please choose how much to ask B to pay:

[Slider from $0 to $(XX+YY)]

<u>Responder's decision</u>
If you are chosen to be the Responder, you will decide whether to accept the Proposer's offer. Please choose the highest amount you are willing to pay to accept an offer:

[Slider from $0 to $(XXX+600)]

[Next page – participants make decisions in rounds 2, 3 and 4]

Questionnaire for all participants

How did you make your decisions? Please be specific and give us as much information as you can (minimum 100 characters).

[textbox]

For the following statements, please answer how strongly do you agree or disagree. [5-point scale from “strongly agree” to “strongly disagree”]

1. People should care more about climate change.
2. Climate change should be given top priority.
3. It is annoying to see people do nothing for climate change problems.
4. People worry too much about climate change.
5. The seriousness of climate change has been exaggerated.
6. Climate change is a threat to the world.

Lastly, please answer a few questions about yourself.

We hear a lot of talk these days about liberals and conservatives. Here is a 7-point scale on which the political views people might hold are arranged. Where would you place yourself on this scale (from 1 being extremely liberal to 7 being extremely conservative)?

a) Extremely liberal
b) Liberal
c) Slightly liberal
d) Moderate, middle of the road
e) Slightly conservative
f) Conservative
g) Extremely conservative

What is your sex?

a) Male
b) Female

What is your age? [textbox]

Additional comments, if any: [textbox]

S5. Participant Comments

Comments. We also examined participants' open-ended comments to the question, "How did you make your decisions?". We looked for themes and found that terms referring to the game's history and comparative words were prevalent, which is consistent with the design of the experiment: a two-stage game that invites comparisons of emissions and wealth between countries. We analyzed whether patterns differed across conditions following the example of previous work[1]. For comparative words, we counted in each participant's comment the number of the following words: *country, party, ratio, fair, more, less, amount, number, than*. We chose these words based on previous work on the linguistics of comparisons[2] and on the specifics of the game. (For comparative words, the most frequently used ones were *more*, *amount*, and *country*.) For words that refer to the history of the game, we counted the words: *previous, before, predecessor, responsible/responsibility, team, stage, round*. (For words referring to history, participants most frequently mentioned *team*, *round*, and *stage*.) We included plurals and other forms for each word. We excluded participants who did not write any comments. Figure 3 shows the results. We find that in the History treatment, participants used almost 6 times more words referring to history than in the Baseline, $t(207) = 4.61$, $p < 0.0001$. In contrast, they used a similar number of comparative words, $t(207) = 0.73$, $p = 0.47$. The key difference is that in the history treatment, many participants explained that they divided the costs proportionally to their historical emissions (e.g., "Maximum amount I would pay was based on proportion of mitigation costs generated by my country in the first stage." or "If my team member incurred more cost, I will be willing to pay more"). In the Baseline treatment, we did not see such explanations because participants did not know about predecessors.

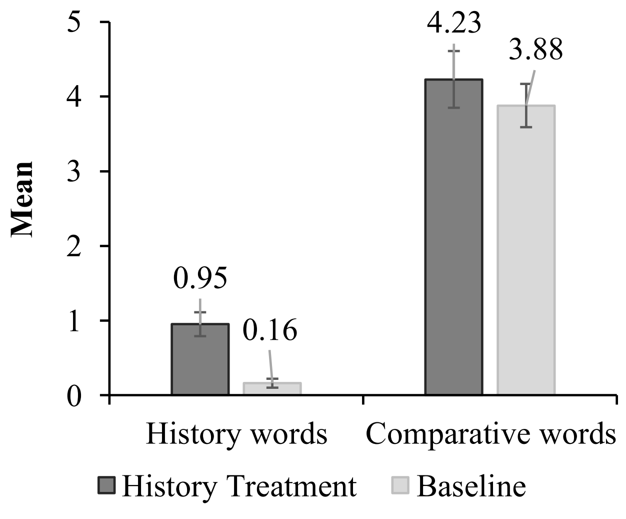

Figure A8. Mean (SE) number of comparative words and words referring to the history of the game depending on treatment (History or Baseline). Comments were provided by 105 participants in the History treatment and by 104 participants in the Baseline. The total number of words related to the history was 100 in the History treatment and 17 in the Baseline. The total number of comparative words was 444 in the History treatment and 404 in the Baseline.

References

1. Del Ponte, A. & DeScioli, P. Pay your debts: Moral dilemmas of international debt. *Polit. Behav.* 1–24 (2021).
2. Kennedy, C. Vagueness and grammar: The semantics of relative and absolute gradable adjectives. *Linguist. Philos.* **30**, 1–45 (2007).
