# Supplementary material for: Information about historical emissions drives the division of climate change mitigation costs
Source: Nat Commun. 2023 Mar 14;14:1408. doi: 10.1038/s41467-023-37130-7 (PMC10012302; doi:10.1038/s41467-023-37130-7)
Supplement: Supplementary file 2 — Reporting Summary [file 41467_2023_37130_MOESM2_ESM.pdf]

nature portfolio

Corresponding author(s): Alessandro Del Ponte

Last updated by author(s): Jan 13, 2023

Reporting Summary

Nature Portfolio wishes to improve the reproducibility of the work that we publish. This form provides structure for consistency and transparency in reporting. For further information on Nature Portfolio policies, see our Editorial Policies and the Editorial Policy Checklist.

Statistics

For all statistical analyses, confirm that the following items are present in the figure legend, table legend, main text, or Methods section.

n/a	Confirmed	
☐	☒	The exact sample size (*n*) for each experimental group/condition, given as a discrete number and unit of measurement
☐	☒	A statement on whether measurements were taken from distinct samples or whether the same sample was measured repeatedly
☐	☒	The statistical test(s) used AND whether they are one- or two-sided<br>*Only common tests should be described solely by name; describe more complex techniques in the Methods section.*
☒	☐	A description of all covariates tested
☐	☒	A description of any assumptions or corrections, such as tests of normality and adjustment for multiple comparisons
☐	☒	A full description of the statistical parameters including central tendency (e.g. means) or other basic estimates (e.g. regression coefficient) AND variation (e.g. standard deviation) or associated estimates of uncertainty (e.g. confidence intervals)
☐	☒	For null hypothesis testing, the test statistic (e.g. *F*, *t*, *r*) with confidence intervals, effect sizes, degrees of freedom and *P* value noted<br>*Give P values as exact values whenever suitable.*
☒	☐	For Bayesian analysis, information on the choice of priors and Markov chain Monte Carlo settings
☒	☐	For hierarchical and complex designs, identification of the appropriate level for tests and full reporting of outcomes
☐	☒	Estimates of effect sizes (e.g. Cohen's *d*, Pearson's *r*), indicating how they were calculated

Our web collection on statistics for biologists contains articles on many of the points above.

Software and code

Policy information about availability of computer code

Data collection	All experiments were conducted online using Qualtrics. Participants were recruited using ORSEE 3.0.5.
Data analysis	The experimental data were analyzed using Stata 14.

For manuscripts utilizing custom algorithms or software that are central to the research but not yet described in published literature, software must be made available to editors and reviewers. We strongly encourage code deposition in a community repository (e.g. GitHub). See the Nature Portfolio guidelines for submitting code & software for further information.

Data

Policy information about availability of data

All manuscripts must include a data availability statement. This statement should provide the following information, where applicable:

- Accession codes, unique identifiers, or web links for publicly available datasets
- A description of any restrictions on data availability
- For clinical datasets or third party data, please ensure that the statement adheres to our policy

The data that support the findings of this study have been deposited in OSF: https://osf.io/5rupw/?view_only=bc4ea055ed8045c896ee9e7b34f57a3c

Human research participants

Policy information about studies involving human research participants and Sex and Gender in Research.

Reporting on sex and gender	Participants were recruited regardless of their gender, therefore the findings do not apply only to one gender. We collected information about sex and gender by asking the participants to report their sex (male or female) in the questionnaire. No sex-based analyses have been performed because the experiment was not designed to study the difference between sexes or genders. Individual data about the sex of the participants is reported in the dataset. On average, the distribution of sexes was as follows: 62% female and 38% male in the History treatment, 69% female and 31% male in the Baseline, 57% female and 43% male in the Baseline with Predecessors.
Population characteristics	See above.
Recruitment	Participants were recruited from an existing database of students who signed up for research participation. The database is managed using ORSEE software. A random subset of the registered participants were invited to sign up for the experiment. The recruitment materials did not provide any specific details about the experiment. When the required number of participants signed up, they were sent a link to the experiment on Qualtrics. Participants self-selected into the experiment. However, they did not know the topic of the experiment when they signed up. Hence, concerns for self-selection bias are allayed.
Ethics oversight	The experiments were approved by the Institutional Review Board at the university where research collection took place.

Note that full information on the approval of the study protocol must also be provided in the manuscript.

Field-specific reporting

Please select the one below that is the best fit for your research. If you are not sure, read the appropriate sections before making your selection.

☐ Life sciences ☒ Behavioural & social sciences ☐ Ecological, evolutionary & environmental sciences

For a reference copy of the document with all sections, see nature.com/documents/nr-reporting-summary-flat.pdf

Behavioural & social sciences study design

All studies must disclose on these points even when the disclosure is negative.

Study description	The data collected in the experiment is quantitative. We performed an economic experiment: participants made their decisions on Qualtrics and were compensated based on their decisions and the decisions of other participants in the game.
Research sample	We recruited participants at a large university in Singapore, aged between 18 and 31 (average was 22). 66% of the participants were female. The sample is not representative. The student sample was used for accessibility, convenience and comparability with the previously published research.
Sampling strategy	Convenience sample. Participants were recruited from an existing database of students, using ORSEE software. Since participants do not observe the decisions of others, all decisions made by any participant are independent of the decisions made by others. We aimed to collect 50 independent observations for each treatment and each player type, therefore we aimed to collect data from around 100 participants per treatment (in the second generation) and an additional 100 to obtain first-generation data. Previous studies on collective-action dilemmas and similar games used 10 groups of 6 players per treatment (ref. #50 and #37), 12 groups of 6 players (ref. #12) or 21-24 groups of 2 players (ref #39), with a total of 42-72 participants per treatment. We collected data from a total of 93-103 participants for each treatment. Our sample size was therefore greater than or equal to comparable experimental studies.
Data collection	Participants who expressed interest in the experiment received a link to the experiment on Qualtrics. Participants had 48 hours to complete the experiment. 120 participants signed up for first-generation experiments and 103 completed the experiment on Qualtrics. 220 participants signed up for the second-generation experiments and 204 completed the experiment. For the additional baseline treatment, 110 participants signed up and 93 completed the experiment. All the participants who received the link and completed the experiment within 48 hours received their earnings and their data was used in the paper. The PI overseeing the experiment was not not blind to the hypotheses but research assistants were. Participants completed the experiments online and had no interaction with the researchers.
Timing	Data collection for the first generation participants ran from 5 November 2020 to 8 November 2020. Data collection for the second generation in the History treatment ran from 13 November 2020 to 16 November 2020. Data collection for the second generation in the Baseline treatment ran from 16 November 2020 to 17 November 2020. Data collection for the second generation in the additional Baseline treatment ran from 31 May 2022 to 2 June 2022.
Data exclusions	Partial responses of participants who started the experiment but did not complete it were excluded from analysis.

Non-participation	17 participants in the first generation and 33 participants in the second generation received a link to the experiment but did not complete it.
Randomization	Data was collected in four waves: first generation experiments, second generation for the History treatment, second generation for the Baseline treatment and second generation for the additional Baseline treatment, requested by one of the reviewers. When participants signed up to take part in a wave, they were not informed about the generation or treatment for which the data is being collected. All participants who signed up for a wave were divided into equal groups and randomly assigned to types (either A1 and B1, or A2 and B2). Participants could take part in only one wave of the experiment. After entering the experiment, each first-generation participant was randomly assigned a productivity condition (equal, slightly unequal or highly unequal). We used Qualtrics to assign the conditions to equal number of participants. Each second-generation participant was randomly assigned four different pairs of first-generation participants.

Reporting for specific materials, systems and methods

We require information from authors about some types of materials, experimental systems and methods used in many studies. Here, indicate whether each material, system or method listed is relevant to your study. If you are not sure if a list item applies to your research, read the appropriate section before selecting a response.

Materials & experimental systems

n/a	Involved in the study	
☒	☐	Antibodies
☒	☐	Eukaryotic cell lines
☒	☐	Palaeontology and archaeology
☒	☐	Animals and other organisms
☒	☐	Clinical data
☒	☐	Dual use research of concern

Methods

n/a	Involved in the study	
☒	☐	ChIP-seq
☒	☐	Flow cytometry
☒	☐	MRI-based neuroimaging
